# SHEA practice update: infection prevention and control (IPC) in residential facilities for pediatric patients and their families

**DOI:** 10.1017/ice.2024.124

**Published:** 2025-01

**Authors:** Judith A. Guzman-Cottrill, Daniel B. Blatt, Kristina A. Bryant, Caitlin L. McGrath, Danielle M. Zerr, Ayelet Rosenthal, Larry K. Kociolek, Catherine Murphy, Karen A. Ravin

**Affiliations:** 1 Department of Pediatrics, Oregon Health and Science University, Portland, OR, USA; 2 Department of Pediatrics, University of Louisville, Louisville, KY, USA; 3 Department of Pediatrics, University of Washington, Seattle, WA, USA; 4 Department of Pediatrics, Northwestern University Feinberg School of Medicine, Chicago, IL, USA; 5 Division of Infectious Diseases, Cincinnati Children’s Hospital Medical Center, Cincinnati, OH, USA; 6 Department of Pediatrics, Sidney Kimmel Medical College at Thomas Jefferson University, Philadelphia, PA, USA

## Abstract

In 2011, the Society for Healthcare Epidemiology of America (SHEA) and Ronald McDonald House Charities® (RMHC®) established a formal collaboration to develop the first IPC guideline. Both organizations agreed that RMH programs staff and other organizations operating similar programs would benefit from a standardized approach. In 2023, the collaboration was re-established to revise and update the original IPC guideline. This SHEA Practice Update on “Infection Prevention and Control (IPC) in Residential Facilities for Pediatric Patients and Their Families” addresses preventing transmission of infectious agents in “home away from home” residential settings, of which the Ronald McDonald Houses (RMHs) serve as a prototype.

## Introduction

Throughout the United States (US) and other countries, families travel long distances to access specialized pediatric care. Ronald McDonald House (RMH) programs, Ronald McDonald Family Room (RMFR) programs, and similar facilities have become integral components of pediatric family-centered care. In this practice update, while we refer to RMH and RMFR programs as the prototype for such facilities, we intend to provide recommendations that are applicable to all facilities of this type. These types of facilities provide support services that include overnight lodging for families with children who are ill or injured. Pediatric *patients* are also frequent guests of the family-centered residential facilities, with their families, while receiving or recovering from specialized medical therapy nearby. Examples include children traveling for hours for a specialty clinic appointment, children who are recovering from organ transplantation and are immune compromised, children receiving intensive outpatient therapy, or premature infants of a multi-gestational birth recently discharged from a neonatal intensive care unit (NICU) whose siblings remain hospitalized. Facilities may also include people who have high-risk pregnancies and must be close to the specialty delivery hospital if labor begins early, or they may nearly exclusively serve families of NICU patients.

In 2011, the Society for Healthcare Epidemiology of America (SHEA) and Ronald McDonald House Charities® (RMHC®) established a formal collaboration to develop the first IPC guideline. Both organizations agreed that RMH programs staff and other organizations operating similar programs would benefit from a standardized approach. In 2023, the collaboration was re-established to revise and update the original IPC guideline.

RMH and RMFR programs provide families with everything from accommodations and meals, social/emotional programming, and holistic support for parents and siblings at little or no cost to guest families while their children receive medical care. In 1974, the first RMHC® family-centered residential facility opened in Philadelphia, Pennsylvania.^1^ As of November 2023, 386 RMH and RMFR programs exist across 62 countries and regions; 184 of these are in the US. An average of 6,078 families stay in a RMHC® facilities per night worldwide. The facilities vary in size, from five to 206 bedrooms per house. Most RMHC® programs are free-standing structures, but some RMHC® programs exist within children’s hospitals. RMH programs provide housing, meals, laundry facilities, and family activities, at little or no cost to occupants. Lengths of stay range from one night to months, depending on the child’s medical needs. There is no maximum length of stay. The minimum criterion for a patient or family to stay includes receiving daily outpatient care or treatment as an inpatient. Families are routinely re-evaluated to determine their eligibility for continuing stay.

RMH programs differ in activities and practices in hospitals and long-term care facilities. RMH programs staff do not provide medical services. Air quality practices are described in the Building Family Centered Spaces Guidelines Toolkit. Standardized policies for environmental disinfection do not exist. While local RMHC® chapters employ staff or service providers to maintain housekeeping and cleanliness standards, RMH and RMFR programs also rely on guests and volunteers for routine cleaning and disinfection of common areas. When a family “checks out” of a sleeping room, the family or a volunteer cleans and disinfects it before the next family “checks in.” Chapter staff, volunteers, or a housekeeping service follow this initial cleaning with a room check to confirm standards of cleanliness.

In all RMH and RMFR programs with overnight accommodations, meals are prepared and consumed in a common kitchen and dining room, and guests share in the clean-up duties after meals. Kitchens are open 24 hours a day, as a child’s around-the-clock medical care and changing health status may dictate a family’s meal schedule. Each family has a designated area to store their own perishable and nonperishable foods. RMH and RMFR programs staff and volunteers review the refrigerator and freezer contents at least once weekly to discard spoiled food. Some houses have rooms or floors dedicated to high-risk transplant patients that include separate food preparation and dining areas.

Guests of RMH and RMFR programs are at risk of exposure to communicable illness in common areas such as family lounges and community kitchens, but it is unknown whether healthcare-associated infections (HAIs), which are infections that patients get while or soon after receiving healthcare, can involve an RMHC® program. It also is not known how guests of RMHC® facilities affect the spread of infections in the affiliated healthcare facility. Anecdotally, pediatric hospital epidemiologists can recall at least one hospital outbreak investigation that included their local RMHC® program, but this has not formally been reviewed. A literature search revealed only one published report (a hospital varicella outbreak involving an RMH).^2^

A local chapter owns and operates each RMHC® program and a locally organized board of directors governs it. All chapters are official sub-licensees of RMHC® Inc., located in Chicago, Illinois. This local governance structure allows for flexibility of policies and practices among individual RMHC® chapters within a framework of global standards, including the management of IPC issues. Most sites have locally written IPC guidelines, and consultation resources vary,^3^ increasing the potential for inconsistency.

Hospital IPC guidelines do not apply to most RMH and RMFR program settings, since these guidelines address practices for patients who are vulnerable when hospitalized, undergoing medical procedures, and have medical devices. It is both unnecessary and unrealistic to expect a family-centered residential facility to adhere to strict hospital policies. Specifically, hospital IPC policies do not align with the purpose of RMHC® to serve families with children who are ill or injured in a *community* setting through its RMH or RMFR program. Development of such recommendations would require a strategy appropriate for the spectrum of RMH and RMFR programs, as they differ in size, amenities, patient populations, and location (free-standing versus a program within a hospital).

Users of this practice update may apply the general and specific topics of IPC to various non-healthcare settings, including concepts of standard precautions, screening visitors for exposures to infectious diseases, excluding staff members and visitors with transmissible infections, prevention of outbreaks, and optimizing the health of local RMHC® staff members and volunteers through education.

This document differs from SHEA-endorsed guidelines. First, the primary audience of this guideline is not healthcare personnel; instead, this is a reference for staff members and volunteers who are educating and monitoring visitors for illness or exposures in a program providing accommodation to children who are ill and injured and their families. The authors chose the terminology used throughout the document for that audience. Second, healthcare epidemiology literature related to this specific pediatric setting is sparse. The authors adapted recommendations based on currently available evidence to this special setting. Where published evidence does not define best practices, the practice update provides practical recommendations developed by the authors and endorsed by SHEA. Recommendations may be modified in cases of outbreaks, novel situations not addressed in this document, or deemed as necessary for safety. Management decisions must be individualized for the specific circumstance, but many recommendations for IPC in day care settings and ambulatory clinics are applicable to RMH and RMFR programs, and similar facilities.

## Background

### Methods

#### IPC needs assessment

Needs assessments were critical steps in guideline development to determine the topics and recommendations included in this document. The assessments (2011, 2023) sought to identify the populations served, the most frequent IPC situations encountered by RMH and RMFR programs’ staff, and the methods and resources used to manage IPC situations. The survey findings are summarized in detail elsewhere.^3^

#### Focus group and site visits

The RMHC® Conference in Chicago (2011 International, 2023 Regional with Chapters from the Americas) held two focus groups. In January 2012, two authors toured three RMH programs in Houston, TX with an RMH programs organizational leader and program managers. These activities provided greater insight regarding the IPC challenges, the variety of physical structures of RMH programs, and the types of questions staff members have about management of patients and families with an infectious condition or a history of exposure to others with infections.

#### Scope

The scope of this update is specifically for family-centered residential facilities that provide accommodation and other services to families with children who are ill and injured. However, these facilities may vary in the populations served, whether healthcare services are provided (RMH and RMFR programs do not provide healthcare services), human and fiscal resources, and governance structure. In such situations, adapt the recommendations to meet these specific conditions. While this guidance was developed for facilities providing pediatric family-centered care, it could be adapted for facilities serving adult patients and their family members and caregivers.

If a family-centered residential facility is located within a medical facility, then the facility’s IPC-related policies and procedures will supersede the recommendations contained in this practice update. In most situations, a hospital policy or procedure will contain more detailed and structured instructions than this document provides.

This document provides recommendations to prevent the spread of infections in RMH and RMFR programs. The authors did not include recommendations to prevent infections caused by pathogens (germs) that are rare, especially for children, or for which evidence is emerging, for example, *Candida auris*. RMH and RMFR programs should direct questions about germs that are not discussed in this document to infection prevention and control experts in the affiliated medical facility.

## Infection prevention and equity

Health equity is the state in which everyone has a fair and just opportunity to attain their highest level of health.^4^ Working toward health equity requires consideration of social determinants of health (SDOH), which are non-medical factors that influence health outcomes.^5^ Infection prevention recommendations are designed to protect children and families but may also have undesired effects that may not be equitable. These negative effects may disproportionately affect families who are already experiencing oppression, bias, or marginalization. For example, an adult caregiver living at RMHC® program who develops influenza and needs to temporarily relocate away from the family-centered residential facility while ill may have trouble accessing meals if the source of the meals and food preparation space was in the facility, and if hospital-based supports for caregivers are not available. Access to meals, housing, and transportation may be more challenging for caregivers and families based on their distance from home and support system, language used, and financial resources. Equity impact assessments (EIAs) and health equity impact assessments (HIAs) are emerging tools that organizations and policy makers can utilize to systematically identify inequitable impacts from policies and identify those that could be given priority for limited resources.^6,7^

Some strengths of RMH and RMFR programs include connectedness to the local community and resources and knowledge of the local community’s needs, and thus each RMHC® program may recognize unique needs and subsequent solutions to support families.

### Recommendations

1. Consider the use of an equity impact assessment/health equity impact assessment (EIA/HIA) when developing and updating the family-centered residential facility’s policies related to infection prevention.
2. Consider strategies to provide language-concordant information about infection prevention policies, such as working with hospital partners to provide translated documents or interpretation services for policy discussions.
3. Develop plans to help to support families that require temporary relocation away from RMHC® program due to infection prevention concerns. Include considerations for what internal or external resources may be available, such as those related to housing, transportation, and meals.
4. Work with hospital and community partners to provide guests of an RMHC® program and their families with resource guides for options for where adult caregivers may be able to seek medical services for testing and treatment of conditions with potential infection prevention implications.

## Annual staff member and volunteer infection control education

All RMH and RMFR programs staff and volunteers should receive IPC education, ideally upon hire then annually. This checklist outlines the recommended topics and areas for infection prevention and control training for RMH and RMFR programs’ staff and volunteers. The training aims to provide the knowledge and skills necessary to maintain a safe and healthy environment for residents, staff, volunteers, and visitors:

1. Basic infection prevention and control principles:
  - Understanding the importance of infection prevention and control in RMH and RMFR settings
  - Introduction to standard precautions (see **Standard Precautions** section below)
2. Hand hygiene:
  - Importance of hand hygiene and when to perform it
  - Proper handwashing technique using soap and water or cleaning hands with an alcohol-based hand sanitizer (ABHS)
3. Masking:
  - Understanding types of masks
  - Recognizing situations that require the use of surgical masks
  - Proper mask usage: how to wear, remove, and dispose of masks correctly
4. Respiratory hygiene and cough etiquette:
  - Educating employees and others on the importance of respiratory hygiene, such as covering the mouth and nose with tissues or the elbow when coughing or sneezing
  - Encouraging employees and others to stay home when experiencing respiratory symptoms to prevent the spread of infections
5. Cleaning and disinfection:
  - Knowledge of appropriate cleaning and disinfection practices for different surfaces and areas within RMH and RMFR facilities
  - Proper and safe use of disinfectants and understanding contact time requirements
  - Regular cleaning schedules and techniques for frequently touched surfaces and shared spaces
  - Appropriate cleaning and disinfection of toys
6. Food management:
  - Understanding food safety principles for prevention of foodborne illness
  - Guidance on the proper handling and storage of food
7. Outbreak management: Familiarity with the facility’s outbreak management protocols and reporting mechanisms

## Core principles of infection prevention and control

### Background

Preventing transmission of infectious agents among patients, families, and healthcare personnel is a challenge in all healthcare settings. Scientific research reviews are used to support the recommendations made for preventing the transmission of infectious agents in healthcare facilities in various guidelines and similar publications by the Healthcare Infection Control Practices Advisory Committee/Centers for Disease Control and Prevention (HICPAC/CDC),^8^ SHEA,^9^ and the Infectious Diseases Society of America (IDSA).^10^ There has been little research and few published guidelines for infection transmission in ambulatory settings and in family-centered facilities. Special ambulatory settings for which IPC guidelines do exist include hemodialysis centers^11^ and cystic fibrosis clinics.^12^ In 2011, CDC developed guidelines for general outpatient clinics^13^ and oncology clinics^14^ that describe the minimum expectations for safe care in outpatient settings. There is no research to inform recommendations to prevent transmission of infectious agents in family-centered facilities that serve as a “home away from home.”

Although RMH and RMFR programs are not healthcare facilities, they are “residences” with potential for exposure to infectious diseases. Patients with medical conditions that make them particularly vulnerable to infection live and share common facilities with many other children and their families. **Communal living can provide opportunities for transmission of infectious agents. By understanding the basic principles of infection prevention, all individuals who live and work in these facilities can reduce the risk of infection for children and families whom they serve.**

### Standard precautions

Standard precautions are a set of practices aimed at preventing the spread of infectious agents. Standard precautions are based on the principle that all blood, body fluids (e.g., material coughed up, spit), secretions, excretions (e.g., urine, stool, wound drainage, but *not* sweat), non-intact skin, and mucous membranes *may* contain transmissible infectious agents. Therefore, containing these fluids will reduce the risk of transmission of infectious agents. Employees, volunteers, patients, family members, and visitors must partner to prevent transmission of infections. Recommendations for Standard Precautions are based on strong evidence from healthcare settings that has been summarized and referenced in guidelines published by CDC.^8,15^

Elements of Standard Precautions can be applied in RMH and RMFR programs, and similar facilities:

1. *Hand hygiene*: This is a general term that applies to either *hand washing* with soap and water, or *cleaning hands* with an alcohol-based hand sanitizer containing at least 60% alcohol. Performing hand hygiene at the appropriate times is a critical step one can take to prevent transmission of infectious agents and has been shown to be effective in both healthcare and non-healthcare settings such as day care centers, dormitories, and schools.^16–19^ Placement of hand sanitizer dispensers at entries to the family-centered residential facilities, in hallways, in guest rooms, in areas where food is prepared, served and consumed, and in areas where there may be contact with animals will promote hand hygiene by all staff, guests, and visitors. Hand washing with soap and water is preferred before eating, after handling soiled diapers or items contaminated with stool, and whenever hands are visibly soiled.
2. *Glove use:* Wear disposable gloves for additional protection when individuals are likely to have contact with body fluids, non-intact skin, and mucous membranes. Gloves are not a replacement for hand hygiene. Gloves reduce the level of contamination on hands, but they do not eliminate it because there may be tiny holes that are not obvious. Perform hand hygiene after removing gloves, since hands can become contaminated during removal. Also, a person should use gloves only a *single* time and never wash and reuse them. In healthcare settings, personnel clean hands both before and after glove use. This practice is ideal for a home setting, but at minimum people in this setting should wash hands immediately after glove removal.
3. *Respiratory hygiene/cough etiquette:*
  - Since it is not always known if a person is contagious when they have respiratory symptoms including cough, congestion, rhinorrhea (runny nose), sneezing, or increased production of respiratory secretions, all respiratory secretions are potentially infectious. A person should cover the mouth and nose with a tissue when coughing or sneezing, immediately dispose of the tissue in a waste receptacle, and perform hand hygiene when finished. If tissues are not readily available, then sneezing or coughing into one’s sleeve or elbow is safer than sneezing or coughing into the air, although practice caution if an infant or child may be held in one’s arms and encounter soiled sleeves.
  - Many viruses and bacteria are transmitted through the air.^20^ Sometimes germs are coughed or sneezed into the air and fall directly on the eyes, nose, or mouth of people nearby. Sometimes germs are coughed or sneezed into the air and may travel just a few feet or longer distances before they are breathed in by other people. A common and practical suggestion is that vulnerable patients should remain at least 3–6 feet (1–2 m) away from individuals who are coughing or sneezing. This may not prevent all infections. The medical team will advise patients with weakened immune systems to avoid crowded places and to wear masks when they are in public areas for additional protection against respiratory tract infections. These “surgical masks,” sometimes referred to as “isolation masks,” cover the mouth and nose, but do not fit tightly on the face. If someone is coughing near a child who is vulnerable, move the child away from the coughing person, or ask the coughing person to move away from the child and to please cover their cough for the protection of others.
4. *Blood and body fluid precautions:* Some infectious agents can be acquired from exposure to blood and body fluids. Staff, volunteers, and guests should always assume that any blood or body fluid may be infectious.
  - Advise families to not share personal items such as toothbrushes or razors with others, even within their own family.
  - Restrict entrance of an individual with open skin infections that cannot be covered or other conditions that might allow contact with their body fluids.
  - If an infected person or their family member has behaviors (e.g., biting) that might put others at risk of contact with their blood or body fluids, specific restrictions may be required, determined on an individual basis.
  - See Section 7 Cleaning and Disinfection of the Environment for guidance on cleaning surfaces contaminated with blood.
5. *Safe injection practices:* Caregivers may need to use needles to administer medications either through a central venous catheter (often called “central line” or “PICC line”) or by direct injection, (e.g., insulin). Because a person’s blood contaminates the needle after injection, a caregiver or program staff member could acquire a bloodborne infection if they sustain an accidental needlestick with a used needle. Hepatitis B virus, Hepatitis C virus, and human immunodeficiency virus (HIV) can transmit via needlestick injuries. Carefully manage all needles used for medication administration. Never re-cap used needles and syringes and never use needles or syringes more than once. When possible, use single dose vials of medications instead of multi-dose medication vials to reduce the risk of contamination. Only one single person should use a medication vial, insulin pen, or finger-stick device for blood sugar monitoring. Always store these medical supplies and devices in the guest’s private room. Dispose of needles separately from all other trash in a rigid, puncture-resistant container. Instruct housekeeping staff and volunteers to exercise caution when cleaning linens and cleaning these rooms. Facilities should have a process in place for employees and staff who sustain a needlestick injury. Additional information about safe injection practices may be found on CDC website.^21^
6. *Safe Laundry Management:* As soon as possible, remove any linens or clothing soiled with blood, stool, or vomitus to prevent transfer of infectious agents to others or the environment. In addition, to prevent the spread of infectious agents from contaminated linens and clothing:
  - Do not shake soiled linens or clothing.
  - Place soiled linens and clothing in a plastic bag to completely contain fluids.
  - Separate soiled linens and clothing from other laundry. Wash soiled linens or clothing in hot water with detergent as soon as possible. At least water temperature of at least 71 degrees Celsius (160 degrees Fahrenheit) for a minimum of 25 minutes. Chlorine bleach can provide additional safety.^22^
7. *Cleaning and disinfection of the environment:*
  - Microorganisms can spread by contact with an infected person (direct spread) or by touching an object or a surface contaminated with infectious secretions or body fluids (indirect spread).^8,22^ Many bacteria and viruses can survive for prolonged periods of time on environmental surfaces. For example, influenza virus can survive on hard surfaces such as stainless steel or plastic for a few days.^23^ Some bacteria can persist on surfaces for months.
  - Cleaning and disinfecting objects and surfaces can help prevent the spread of infection in family-centered residential facilities. The American Academy of Pediatrics (AAP) offers a reference guide which includes recommendations for routine environmental cleaning and disinfection in schools and daycares, which can be adapted for residential settings.^24^
    - Cleaning involves removal of dirt and surface contamination, usually by scrubbing with a detergent and then rinsing with water. Cleaning is an especially important first step because visible substances (e.g., stool, nasal discharge) reduce the action of disinfectants. Follow routine housekeeping procedures (e.g., cleaning, wet mopping, dusting, and vacuuming) to reduce the spread of infection in facilities such as childcare centers and schools.
    - Sanitizing reduces pathogens on inanimate surfaces to levels specified by public health authorities or regulations deemed as safe.
    - Disinfection destroys or inactivates most germs (except spores) on objects or surfaces and is especially important for food preparation surfaces.
  - Have a procedure in place for routine cleaning and sanitizing or disinfecting environmental surfaces in common areas that specifies:
    - Who is responsible for cleaning and disinfection?
    - What areas and items should be cleaned and disinfected?
    - Frequency of cleaning and disinfection
    - Who is responsible for supervising and inspecting areas that have been cleaned?
    - Frequency of inspections
    - Documentation of procedure completion and compliance
  - More frequent cleaning/disinfection may be required during outbreaks
  - For areas that require disinfection, such as toilet and diapering areas and food preparation areas, use hospital-grade germicides. The National Resource Center for Health and Safety in Childcare and Early Education has guidance for selection and use of a cleaning, sanitizing, or disinfecting product that could be adapted by residential facilities.^25^ Alternatively, the CDC has published guidance for cleaning and disinfection in early care and education setting that could serve as a resource for residential facilities.^26^
  - Follow manufacturers’ instructions for use when mixing liquids for disinfecting floors and surfaces. When Environmental Protection Agency (EPA)-registered disinfectants are not available, a dilute bleach solution may be used. CDC provides recommendations for safely mixing and using bleach. Key points include but are not limited to:
    - Follow the label directions on the bleach product.
    - Check to see if protective equipment, such as gloves or eye protection, is needed
    - Use regular, unscented household bleach containing 5%–9% sodium hypochlorite. Do not use a bleach product if the percentage is not in this range or if it is not specified on the label
    - Follow the directions on the bleach bottle for preparing a diluted bleach solution. If your bottle does not have directions, a bleach solution can be made by mixing 5 tablespoons (1/3 cup) of bleach per gallon of room temperature water
    - Always follow the manufacturer’s instructions for applying the bleach solution to surfaces. If instructions are not available, leave the diluted bleach solution on the surface for at least 1 minute before removing or wiping the surface
    - Make a new diluted bleach solution daily. Bleach solutions will not be as effective over 24 hours after being mixed with water
    - When bleach is used for disinfection after large blood spills, a more concentrated solution is used (see e.g. below)
  - Disinfect the surface of a blood or other potentially infectious body fluid spill with an EPA-registered disinfectant labeled to kill tuberculosis (“tuberculocidal”) or a bleach solution. A more concentrated bleach solution of 1 part bleach to 9 parts water is used with large blood spills and when disinfecting the room of a guest with recent *C. difficile* infection (CDI). Immediately clean spills of blood and other potentially infectious body fluids using disposable towels, wearing disposable gloves. Dispose of blood-contaminated materials in a plastic bag closed with a secure tie.
  - When using EPA-registered products, adhere to contact or “dwell” time (the length of time a solution must be in contact with a visibly clean surface to kill germs) recommended for each product. Some products may require several minutes of contact time to be effective. The surfaces should stay wet during the entire contact time to make sure that germs are killed.
  - The US Occupational Safety and Health Administration (OSHA) requires that employers provide their workers with the Safety Data Sheet for any cleaning products employees should use. All chapters should comply with local regulations regarding training in employees’ use of cleaning products.
  - For floors, rugs and carpeting contaminated by body fluids: While wearing gloves, immediately blot to remove excess fluid, then spot-clean the area with a detergent-disinfectant. Shampooing or steam cleaning may be required.
  - In common areas and playrooms, choose toys that can be easily cleaned and disinfected (avoid stuffed or cloth toys because they are not easily cleaned). Clean and disinfect toys daily or more frequently when toys are contaminated with mouth or nose secretions. Small plastic toys may be cleaned and disinfected in a mechanical dishwasher if dishes are not washed at the same time.
  - To reduce the potential for environmental contamination with fecal matter, instruct families to perform diaper changes in a guest’s room or the bathroom (or other clearly designated area). Diaper changes should not take place in common areas. Place soiled diapers in plastic lined, lidded designated receptacles remote from food preparation areas. Regularly clean and disinfect diaper changing tables in communal bathrooms (at minimum once daily or whenever visibly soiled). Never use sinks intended for hand washing or food preparation for rinsing soiled clothing or linens, for cleaning equipment used in toileting, or for disposal of cleaning wastewater.
  - Provide education at least annually for staff responsible for cleaning, sanitizing and disinfecting. At a minimum, the training should include:
    - How to handle, mix, and store solutions
    - When to use personal protective equipment, including gloves
    - Information required by OSHA about the use of any chemical products
    - Disposal of materials contaminated with blood and body fluids

## Food safety

### Food poisoning

Some foods more commonly cause food poisoning than others, including raw or undercooked meat and poultry, raw seafood, raw or undercooked eggs, raw (unpasteurized) milk and soft cheeses made from raw milk, raw flour, and uncooked or unwashed fruits and vegetables. A variety of germs can contaminate these foods and cause illness, such as norovirus and bacteria such as *E. coli* and *Salmonella*. CDC estimates that 48 million people in the US become ill due to foodborne infections every year. These infections can spread directly from person-to-person and through contaminated foods.

Depending on the germ causing the illness, symptoms may appear within 30 minutes to up to 7 days after ingestion. Typical symptoms of food poisoning include nausea, vomiting, cramps or abdominal pain, diarrhea, and may also include fever. In mild cases, symptoms last for 24–72 hours. In more severe cases, vomiting and diarrhea may be prolonged, people may become dehydrated due to a lack of adequate fluid intake, diarrhea may be bloody, and fever may be higher than 39 degrees Celsius (102 degrees Fahrenheit).

Anyone can get food poisoning. Most people who do have mild cases. However, some people are at higher risk of more severe disease. These higher risk individuals are those whose immune systems are not functioning normally. Individuals in this category include children under 5 years old, individuals who are pregnant, adults aged 65 years or older, anyone who may be receiving immunosuppressive treatments, such as cancer or transplant patients, and patients with HIV/AIDS, diabetes, or liver or kidney disease.

### Foodborne illness prevention

#### Food preparation

To reduce the chance of people getting food poisoning, the US CDC and Food and Drug Administration (FDA) recommend 4 simple rules: clean, separate, cook, and chill (see Supplementary Material, Figure 1).^27,28^

#### Clean

Rinse fresh fruits and vegetables under running water. Wash your hands before, during, and after meal preparation, especially when handling uncooked meat, poultry, seafood, eggs or flour. After the meal is prepared, wash all used utensils and cutting boards with hot, soapy water either by hand or in a dishwasher. Clean with disinfectant all countertops and other soiled surfaces. Choose a disinfectant marketed for use on kitchen or bathroom surfaces per the disinfectant brand’s instructions. Wash your hands before eating.

#### Separate

Keep uncooked meat, poultry, seafood, and eggs contained apart from ready-to-eat foods when stored in the refrigerator and while being handled during meal preparation on cutting boards, plates, etc. Use appropriate containers to keep juices from raw foods from contacting ready-to-eat foods.

#### Cook

Cook foods to specific internal temperatures to kill any contaminating germs (see Supplementary Material, Table 1). Cook microwaved food to an internal temperature of 74^o^C/165^o^F. Use food thermometers inserted midway into the food to ensure food has reached appropriate internal temperatures.

#### Chill

Most bacteria multiply more rapidly at room temperature. To help prevent foodborne illness, refrigerate uncooked perishable food like meat, poultry, seafood, eggs, dairy, fruit, and vegetables, as well as cooked leftovers within 2 hours of being at room temperature. Keep refrigerators at 4^o^C/40^o^F or below. Keep freezers –18^o^C/0^o^F or below. Discard all foods before they are likely to spoil (see Supplementary Material, Table 2).

Of note, in this section, our primary focus is on preventing foodborne illnesses from contaminated food products. However, we also emphasize the importance of preventing the transmission of other infectious diseases while handling food for others. Individuals exhibiting respiratory symptoms such as coughing or sneezing, as well as gastrointestinal symptoms like vomiting or diarrhea, may inadvertently contaminate food during preparation and potentially pass on infectious illnesses to others. Therefore, it is strongly recommended that individuals involved in food preparation do not have symptoms of illness, as outlined in Appendices B1 and B2.

## Masking

Masking is an important infection prevention and control method that can significantly reduce the risk of transmission of infectious agents, including respiratory viruses. Masking helps to prevent the spread of respiratory droplets (particles) that may contain germs, even among individuals who do not have symptoms of illness. Commonly used masks^29,30^ include:

1. Surgical masks (also called procedural masks): disposable, loose-fitting masks that cover the nose, mouth, and chin. These are the most commonly used masks in residential facilities for pediatric patients and their families. Surgical masks help protect wearer against exposure to respiratory droplets, and those interacting with the wearer from being exposed to the wearer’s respiratory droplets, which can contain viruses that cause respiratory infections such as the common cold and influenza.
2. N95 respirators: tight-fitting, filtering respirators that block respiratory droplets and efficiently filter airborne particles, used to protect from infections such as COVID-19 or tuberculosis.
3. KN95 masks: similar to N95 respirators in terms of their filtration capabilities.
4. Respirators with exhalation valves: allow for easier exhalation and reduce heat buildup inside the mask. While they protect the wearer, exhalation valves are not suitable for protecting others from the wearer, as they release potentially infectious respiratory droplets into the environment. They generally are not recommended for use in healthcare or public settings.
5. Cloth masks: reusable masks made of various fabrics. They typically have multiple layers to enhance filtration. Cloth masks can help reduce the spread of respiratory droplets from the wearer to others. They may offer some level of protection to the wearer as well, depending on the fabric’s filtration properties and the fit of the mask. People in community settings commonly use cloth masks, but these usually are not recommended in a healthcare setting.

The specific recommendations for mask wearing in RMH and RMFR programs may vary depending on factors such as local regulations, whether specific respiratory germs are spreading in the community, guidance from public health authorities, and patient-specific risk factors. Consult with local public health and/or infectious diseases experts. General recommendations regarding face masking in RMH and RMFR programs include:

1. Guests with respiratory symptoms should restrict themselves to their private room until all symptoms resolve (e.g., runny nose, cough, and congestion).
  - If there is a need to pass through a common area, such as lobbies or hallways, these guests should wear surgical/procedural masks.
  - If procedural/surgical masks are not available, use multilayer cloth masks made from tightly woven fabric.
  - Because RMH and RMFR programs do not accommodate individuals with active highly transmissible infections such as COVID-19 and tuberculosis, they generally do not require staff to wear N95 or KN95 masks. However, staff may wear additional respiratory protection with N95/KN95 based on personal risk factors for infection, personal preference, and local epidemiology of respiratory illness. Some healthcare facilities may advise patients who are immune compromised to wear N95 or KN95 respirators.
2. If possible, ensure an adequate supply of masks is available for staff, patients, and families.
3. Ensure competency and educate individuals in proper mask usage, including how to wear (cover the nose and mouth), remove, and dispose of masks correctly, as well as maintaining hand hygiene practices while wearing masks. Surgical masks should be disposed of at the end of the day. Discard a mask that is damaged, wet, soiled, or is difficult to breathe through.
4. RMH and RMFR programs’ leaders should follow the most evidence informed national or local health authorities’ specific guidelines and instructions regarding masking in common areas. Consult the partner hospital on its current masking policies.
5. There are specific instances where mask usage may be contraindicated or less suitable. Children under the age of 2 years and individuals with physical, developmental, or behavioral impairments that make wearing a mask unsafe should not wear a mask. It is important to consider individual circumstances and consult with healthcare professionals or follow local guidelines for precise recommendations.

## Animals

Animals can be a source of various germs that cause infections in humans. Thus, family-centered residential facilities should ensure that animals allowed facility entry are healthy. Facilities should be aware of local and federal laws related to service animals. To minimize the risk of animal-related infections, recommended precautions include:^31^

1. Do not allow reptiles or rodents (including turtles, mice, hamsters, gerbils, and rats) into the family-centered residential facility.
2. Appropriately maintain fish tanks, if present. Fish tanks should be covered to prevent water splashing into the surrounding air and from guests placing their hands in the tanks. Do not allow guests to play with fish tank water.
3. Ensure that service animals or pets are healthy prior to facility entry by requiring written documentation.
4. If service animals or pets are present, perform hand hygiene both before and after touching the animal.
5. Do not allow service animals or pets to lick guests who have medical devices or compromised immune systems.
6. Notify immune compromised guests if an animal is present in the RMHC® program. Request the family to consult the patient’s clinical team to determine if the patient should avoid the animal.

## Health screening of house guests and visitors

Systematically screen all visitors and prospective guests for illness or exposure to infectious diseases when they arrive at the reception desk or when guests arrange to stay. The healthcare facility, RMH, or RMFR staff can perform the screening process, which should include a standardized list of questions about symptoms and exposures. The authors have included a modifiable health screening tool (see Supplementary Material, Appendix B, Algorithm B1) to fit the needs of each family-centered residential facility. Each facility should develop a standardized method and train staff to follow the same procedure.

Reschedule a visitor if they answer “yes” to any of the questions in the screening. Similarly, arrange for alternative accommodations for prospective guests who answer “yes” to any of the questions, or have these guests remain in their private room, depending on the specific situation (see Supplementary Material, Appendix C, Table C1). Since hospital social workers are frequently involved in the family screening and referral process, make them aware of the screening process details.

If allowed per local governing bodies, ask each prospective guest about his or her measles or varicella (chickenpox) immune status, as well as other vaccine preventable diseases. Maintain information on-file until the guest checks out from the family-centered residential facility, always taking steps to maintain the confidentiality of this information. Families can check with their primary care physician or licensed clinician to see if they are up to date with vaccines. Rapid access to this information is beneficial in case a facility is involved in a measles or chickenpox outbreak investigation. During respiratory virus season, ask prospective guests about their influenza and COVID-19 vaccination status. If the family member has not yet received vaccines, facilities should assist guests to access influenza and COVID-19 vaccines (e.g., nearby pharmacies, medical clinics, and public health clinics) whenever possible. Refer to the WHO childhood and adult immunization recommendations.^32^

Do not automatically exclude from services family members of a patient diagnosed with an infectious disease. Program staff members should refer to the “Specific Diseases and Pathogens (Germs)” section for details. Some specific diseases can cause outbreaks and require a signed medical clearance document that confirms the family members are not an infection risk to guests, staff, and volunteers. A clinician who is familiar with the child’s medical condition should complete the document. Each organization operating a family-centered residential facility should use a standardized document so that all staff can easily recognize a properly completed form. We have provided a Sample Medical Clearance Form (see Supplementary Material, Appendix A, Figure A1). Do not refer to or allow facility entry of a family if they are unable to obtain a signed medical clearance form or if the form is incomplete.

To reach a final management determination if a situation arises where this document does not provide a clear recommendation or if the involved medical clinician and program staff are uncomfortable with a recommendation, seek the opinion of the program manager, involved medical team, and/or other local leaders (such as an affiliated infection control program or public health department). Local consultants may recommend more stringent infection control measures when there is evidence of ongoing transmission or concern for an outbreak.

## Staff member and volunteer illness and vaccination

Staff may pose an infectious risk to co-workers, guests, and visitors. All family-centered residential facilities should develop policies and procedures for evaluating and excluding ill staff and volunteers. At a minimum, staff members and volunteers should report their symptoms of any potentially contagious infection (e.g., respiratory or gastrointestinal tract illnesses; vaccine-preventable diseases including chickenpox, measles, and mumps) to their supervisor. A licensed clinician may need to conduct an evaluation to determine ability to work.

Ideally, all program staff members and volunteers should routinely consult with their primary care physician or licensed clinician to verify their immunizations are up to date, as allowable per local governing bodies. When staff members and volunteers are up to date on all recommended vaccines, they lower the risk of exposure of vulnerable patients and their families to vaccine-preventable illnesses, such as influenza, COVID-19, pertussis (whooping cough), measles, mumps, and chickenpox. See CDC and WHO adult routine vaccination recommendations for more detailed recommendations.^32,33^

## Breast milk storage and maintenance of breast pumps

The AAP recommends exclusive breastfeeding for all infants for approximately the first 6 months of life,^34^ with continued breastfeeding until 2 years or beyond if desired by the mother and child. Breastfeeding or the provision of human milk offers benefits to the parent who is breastfeeding and the child. Mothers of infants who are hospitalized, or mothers who must be separate from their infants because of the hospitalization of another child, may need to express and store breast milk while staying as a guest at a family-centered residential facility. Human milk is not sterile and proper storage and handling reduces the risk of contamination and growth of microbes that could cause illness. Proper storage and handling of breast milk is a key component of a facility’s food safety program.

If the RMHC® program provides shared breast pumps, encourage individuals who are breastfeeding and using the pumps to follow CDC recommendations for cleaning and sanitizing pump kits,^35^ cleaning the pump’s dials, power switch, and adjacent countertop with a disinfectant wipe between uses. Store expressed breast milk in glass or food-grade plastic containers with tight-fitting lids, or in plastic bags designed specifically for human milk storage. Clearly label each container with the infant’s and mother’s full names and the date and time the mother expressed the milk. Store breast milk so that guests have access only to milk that belongs to their infant(s). Keep breast milk in a refrigerator in a guest’s room or in a separate, clearly labeled and preferably locked container in the communal refrigerator. Freshly expressed breast milk may be refrigerated safely at 4^o^C (39.2^o^ F) or colder for up to 96 hours.^36^ Milk may be stored in the freezer [**0°F (**–**18°C) or colder)] and** optimally used within 6 months, and up to 12 months. Keep thawed breast milk refrigerated and use within 24 hours.

Inadvertent administration of expressed breast milk to the wrong infant has occurred in healthcare and daycare settings and could theoretically cause exposure to infectious agents, including HIV, cytomegalovirus, and hepatitis B, although the risk of transmission of infectious diseases is small.^37^ In healthcare settings, accidental exposure to breast milk that is not from the infant’s mother is generally managed in the same manner as accidental exposure to blood.^38^ Guidance from CDC outlines the steps to follow if a child is inadvertently fed another child’s expressed breastmilk.^38^ Notify the mother who supplied the breast milk (donor mother) that her milk was administered inadvertently to the wrong child. Document the mother’s answers about how the milk was expressed and handled before being stored at the family-centered residential facility.

Inform the parents of the exposed infant that the risk of HIV transmission is low from expressed breast milk because individuals who are HIV-positive are advised not to breastfeed their infants if the viral load is detectable.^37^ Transmission of HIV from a single breast milk exposure has not been documented. Although hepatitis B surface antigen has been detected in the breast milk of individuals who are infected with hepatitis B-infected, breastfeeding is not thought to significantly increase the infant’s risk of infection, particularly when a birth dose of hepatitis B vaccine has been given. The risk of hepatitis B transmission from an inadvertent breast milk exposure is low. Hepatitis C is not transmitted through breastmilk. Although some maternal medication passes into breast milk, the risk of adverse effects in a child after a single feeding is very low. Refer the child who received the incorrect breast milk to their primary care physician or licensed clinician for further guidance and to discuss the need for any medical management of diagnostic testing.

## Special populations

Healthcare settings identify highly vulnerable *special populations* based on their risk of acquiring infection and the risk of developing more serious disease once infected, as compared with otherwise healthy individuals. These special populations are also likely to spend more time in healthcare facilities (e.g., frequent clinic visits, prolonged hospitalization) due to their underlying conditions. These patients may have medical devices (e.g., central venous catheters, tracheostomy tubes, and dialysis catheters) that make them even more susceptible to serious infection. Patients with weakened immune systems either from a congenital (born with) or acquired immune deficiency, chemotherapy, or immune-suppressive medications may not respond to vaccines or develop protective antibodies after an infection.

Healthcare teams of families of patients with these special conditions have educated them about precautions they should follow to reduce the risk of infection. When a family arranges to stay in a family-centered residential facility, ask if the child’s medical team has recommended restrictions (see Supplementary Materials, Appendix C, Table C2. for information about frequently encountered special populations including newborns, premature infants, individuals living with immune deficiencies or undergoing immune-suppressive therapy, individuals with cystic fibrosis, and individuals who are pregnant).

## Protection of patients from mold spores who are highly immune compromised

Organisms routinely found in soil, water, construction dust, and decaying organic matter, including fungus such as *Aspergillus* species that release spores into the air, may cause serious disease of the lungs, sinuses, brain, and other organs in highly immune compromised patients (e.g., those with very low white blood cell counts). In hospitals, hematopoietic stem cell (bone marrow) transplant patients are the most immune compromised patients and stay in units with special air handling systems and rooms with specialized air flow with respect to other rooms and corridors, called a “Protective Environment.” Once these patients are well enough to no longer require this protective environment, the hospital may discharge them to their home or to a family-centered residential facility. Although these facilities are not medical facilities and are not equipped to provide a Protective Environment for these patients, an RMHC® program may undertake simple measures to reduce the risk of airborne transmission of fungal organisms. These include proper installation and maintenance of heating, ventilation, and air conditioning (HVAC) systems, and year-round scheduled changes of filters according to manufacturers’ recommendations to prevent dust overload.

Family-centered residential facilities should notify medical personnel and guests of planned activities that increase dispersal of fungal spores from the soil, including construction and renovation projects. During construction and renovation, highly immune compromised individuals should consult their primary care physician or licensed clinician about their exposure risks and inquire if they should consider alternative housing.

If a child is highly immune compromised, the healthcare team may recommend additional precautions:

1. Avoid exposure to the outdoors on windy days. This includes outdoor playgrounds and construction sites.
2. Wear a special type of mask (N-95 respirator) that fits tightly on the face when going outdoors for necessary travel to and from the hospital.
3. Avoid carpeting, because carpet can retain mold spores that can be dispersed into the air during vacuuming or other activities.
4. If carpeting is present, vacuum the area regularly, using a high efficiency particulate air (HEPA)-filtered vacuum when the patient is not present.
5. Avoid the use of humidifiers and dehumidifiers in common areas. If the RMHC® program uses humidifiers or dehumidifiers in individual rooms, observe the recommended care practices per manufacturer or as instructed by healthcare staff members.
6. Removed all potted plants or fresh flowers from the family’s sleeping room.
7. Avoid gardening, digging, and spreading mulch or soil.
8. Avoid areas where gardening, digging, and mulching are taking place.

## Specific diseases and pathogens (germs)

### Bed bugs

#### Background

Bed bugs (*Cimex lectularis)* are reddish brown, wingless, insects measuring 1-7 mm in length. During the day, bed bugs hide in the seams of mattresses, bedding, box springs, headboards, under carpets, and behind loose wallpaper and at night they feed on the blood of sleeping humans. Clues of a possible bed bug infestation include a sweet, musty odor in the space and rust-colored blood spots on mattresses or furniture. Complaints of finding itchy skin bumps upon waking should raise suspicion of a bed bug infestation.

Over the past 30 years, reports have increased of bed bug infestations in homes, hotel rooms, apartments, dormitories, and hospitals.^39^ Bed bugs can be carried on suitcases, furniture, clothing, and other personal items. Clothing and luggage can spread bed bugs from one infested room to another because they can be carried on suitcases, furniture, clothing, and other personal items. Bed bugs may also travel short distances (i.e., room to room or apartment to apartment) by migrating along pipes or ventilation ducts.

Bed bug bites often result in itchy red bumps that look similar to or slightly larger than mosquito bites. They usually appear on parts of the body exposed during sleeping. Not all people bitten by bed bugs develop a skin reaction, which is an allergic response.

Bed bugs have not been proven to transmit infectious diseases but occasionally skin that has been scratched will become secondarily infected with bacteria that normally live on the skin.^40^ Individuals with bed bug bites are not contagious.

Practical guidance exists for prevention and management of bed bugs in group living facilities. Guidelines by Gangloff-Kaufmann et al include photos of bed bugs at different life stages and detailed instructions for carrying out inspections.^41^

Successful eradication of bed bugs is best accomplished by professionals who perform a thorough inspection and, if indicated, apply an insecticide. More than one treatment with insecticide may be required.

#### Recommendations

1. If a family-centered residential facility suspects a bed bug infestation, instruct families to inspect bedding, mattresses, luggage, clothing, and other belongings for the presence of bed bugs.
2. If bed bugs are present, the facility should:
  - Notify its affiliated healthcare facility to have it assess relevant inpatient rooms for infestation.
  - Wash and dry clothing and bedding at hot temperatures.
  - Seal items that are not amenable to washing and drying in a plastic bag and remove them from the facility.
  - Consider pursuing professional inspection and evaluation for treatment with insecticide.
  - Perform serial room inspections after taking these measures.
3. If inspection finds one room is infested, the facility should inspect other rooms based on the guidance of a pest-elimination specialist. In some locales, it is typical to inspect rooms above, below, and on either side of a room with a bed bug infestation.
4. Adjunctive control measures include:
  - Vacuuming (when bed bugs have been identified, use a HEPA-equipped vacuum that is dedicated only to pest control)
  - Reducing clutter where bed bugs could hide
  - Eliminating peeling paint and plaster and caulking cracks and crevices in wall and furniture that could harbor bedbugs
  - Encasing mattresses and box springs in protective covers.

### COVID-19

#### Background

The virus SARS-CoV-2 causes coronavirus disease 2019 (COVID-19). The COVID-19 virus spreads through droplets and small viral particles that are breathed in or that land on people’s eyes, noses, or mouths. People who have COVID-19 can spread infection even if they do not have symptoms of illness. The risk of getting COVID-19 is higher when people are exposed to an infected person for over fifteen minutes, at a close distance, when the infected person has symptoms, and in indoor spaces with poor ventilation. The chance of getting COVID is lower when the infected or exposed person is wearing a well-fitting face mask.^42^

COVID-19 can cause a range of symptoms, but most commonly causes respiratory symptoms similar to a cold or flu, such as fever, congestion, cough, and fatigue.^43^ Other symptoms include headache, muscle aches, loss of taste or smell, nausea, vomiting, and diarrhea. Some people who have had COVID-19 can have long-term effects called “long COVID” or post-COVID conditions (PCC).

Most people with COVID-19 have mild symptoms, although COVID-19 can also cause severe symptoms or death. People who are older, have weakened immune systems, or have certain underlying health conditions are more likely to become very ill from COVID-19. Children served by RMH and RMFR programs may fall into higher risk groups.

Vaccination for COVID-19 has been very important in reducing the risk of severe illness, hospitalization, and death. People who are up-to-date with their COVID-19 vaccines, meaning that they have received all the doses for which they are eligible, have a lower risk of severe illness, hospitalization, and death than those who are unvaccinated or have not completed all doses. It is likely that people will continue to be recommended to get doses of updated vaccines.^44^

#### Recommendations

1. Restrict individuals with suspected or confirmed COVID-19 from entering the family-centered residential facility until 5 days from when they were diagnosed with COVID-19 or had their first symptoms (whichever is earlier) and until they have been fever-free for 24 hours without the use of fever-reducing medicine.
2. Instruct a guest to stay in their private room until their symptoms (runny nose, cough, etc.) are resolved.
3. If symptoms resolve within 10 days after their illness began, they should wear a mask that is well-fitting and high quality (e.g., procedural or surgical mask) when indoors and around others (such as in community spaces in the facility) through day 10. It is important to note that recommendations for how long a person should wear a mask may change.
4. Guests who have had close exposure to someone with COVID-19 (e.g., being less than 6 feet away from a person who has COVID-19 for at least 15 minutes) should wear a high-quality mask when around others and test immediately if they develop symptoms. Even if they do not develop symptoms, they should test at least 5 days after their last exposure and continue masking until 10 days after the exposure.
5. Refer any guest who develops an illness suspected to be COVID-19 for medical evaluation. If diagnosed with COVID-19, the guest must leave the facility until they meet the criteria in **1** above.
6. COVID-19 vaccines reduce the risk of severe disease in guests, staff, and volunteers. (Please see “Staff Member and Volunteer Illness and Vaccination” and “Health Screening of Guests and Visitors of RMHC® Programs” above for more details.)
7. Although the risk of getting COVID-19 because of contact with potentially contaminated surfaces is low, facilities should use EPA-registered disinfectants according to their manufacturer’s instructions for use.
8. Facilities should have an ongoing partnership with their affiliated healthcare facility’s infection prevention and control experts and their public health department for current COVID-19 prevention recommendations on the use of masking and other measures, and how these recommendations apply. Recommendations for facilities may differ from those for the general community because of the expected presence of children with underlying health conditions.

### *Clostridioides difficile* diarrhea (“C. diff” diarrhea)

#### Background

*Clostridioides difficile* (commonly referred to as “C. diff” or “*C. difficile*”) is a bacterium that may be found in the intestines of healthy infants and in a smaller percent of otherwise healthy children and adults.^45,46^ *C. difficile* infection (CDI) is one of the most frequent infections that occurs in healthcare^45^ and commonly leads to inflammation of the large intestine (colitis), along with fever, abdominal pain and/or cramping, and diarrhea with or without blood.^46,47^ CDI occurs most often in hospitalized patients who get antibiotics, chemotherapy, or surgery. It also can occur in people who do not have risk factors.^46,47^

*C. difficile* has the ability to survive for extended periods of time on surfaces and on the hands of the people who come into contact with the spores.^45^ Uninfected individuals can acquire *C. difficile* by unknowingly swallowing these spores after coming in contact with them. In healthcare facilities, preventing transmission of *C. difficile* involves avoiding unnecessary antibiotic use, placing patients with CDI on contact precautions (patient cared for in a private room and healthcare personal wear gowns and gloves), washing one’s hands with soap and water after removing gloves used during care, and thoroughly cleaning the rooms of infected patients.^45^ In residential settings, prevention strategies are focused on hand washing and disinfecting surfaces. There is no vaccine available for *C. difficile.*

#### Recommendations

1. Guests and visitors who have or develop symptoms of CDI, such as fever or bloody diarrhea:
  - Should seek medical evaluation. If the medical evaluation shows that a guest may have CDI, the guest must leave the family-centered residential facility.
  - Should not enter or re-enter the facility until they have started treatment with appropriate antibiotics, their diarrhea has resolved, and they have been fever-free for 24 hours without the use of fever-reducing medicine.
  - Once symptoms have resolved, they do *not* need a negative *C. difficile* test to enter or reenter the facility. Best practices discourage repeat testing of *C. difficile* to prove the person is no longer infected.
2. Guests with recent CDI (defined as having had a diagnosis of CDI within one month) should be restricted from food preparation and food handling in the common kitchen area
3. Once they check out from the facility, the surfaces of their room and bathroom should be cleaned with household bleach diluted with water (1 part bleach, 9 parts water).
4. Guests and visitors who have been exposed to someone with CDI but who do not have symptoms of illness:
  1. Should be allowed to enter the facility
  2. If caring for someone with CDI, they should be educated about the importance of consistent use of disposable gloves and washing the full surface of both hands with soap and water for at least 15 seconds after glove removal.

### Conjunctivitis (pink eye)

#### Background

Conjunctivitis is an inflammation of the inside surface lining of the eyelids and covering of the eyeball. The eye may appear red and may have a clear or cloudy discharge. Conjunctivitis has both noninfectious and infectious causes that can be bacterial or viral. Conjunctivitis does not necessarily require treatment with topical or oral antibiotics.

Many large, community-based outbreaks of conjunctivitis, including outbreaks among school children, on college campuses, and in military facilities have been documented.^48–55^ In neonates, conjunctivitis can be associated with serious invasive disease that involves multiple organs.^56,57^ Careful hand hygiene can reduce transmission.

Individuals who have conjunctivitis should be considered contagious until their symptoms have resolved. The viruses and bacteria that most commonly cause conjunctivitis are transmitted to eyes from hands that are contaminated with the infectious eye drainage, from contaminated objects (shared towels and washcloths, eye drop solution, or contact lens solution), or potentially by direct inoculation into the eye via large respiratory droplets from an ill individual who is coughing.

#### Recommendations

1. Restrict individuals with severe conjunctivitis (i.e., copious eye discharge, eye irritation causing frequent eye rubbing or itching, or conjunctivitis accompanied by fever or respiratory symptoms) from entering the family-centered residential facility.
2. Allow individuals with mild conjunctivitis (“pink eye” with no active discharge, minimum eye irritation, and no fever) to enter the facility, but require such guests to stay in their personal room until their symptoms have resolved.
3. Allow individuals who were exposed to a person with conjunctivitis but who do not have symptoms of illness to enter the facility. No special precautions are required.
4. Educate guests and visitors about the importance of frequent hand hygiene, avoidance of sharing personal items (e.g., towels and washcloths, eye drops, and contact lens solutions), and prompt removal and careful handling of contaminated items (e.g., tissues, towels, and linens).

### Cytomegalovirus (CMV)

#### Background

Cytomegalovirus (CMV) is a common viral infection. Symptoms vary by age and some people with the infection may not show any symptoms. People who have symptoms of CMV may have a mild illness with fever, body aches, a sore throat, and/or swollen glands in the neck. CMV can cause a mononucleosis (“mono”), similar to the illness caused by the Epstein Barr virus (EBV).

Most healthy people do not get seriously ill from CMV. In people who are immune compromised, infection can affect multiple organs. When a pregnant person is infected with CMV, the infection can be passed to the unborn baby across the placenta. One in five babies infected with CMV before birth (congenital CMV) may have long-term health problems including developmental and hearing abnormalities.^58^

Most person-to-person transmission of CMV is a result of exposure to infected secretions such as saliva or urine. Children who attend childcare centers can be infected with CMV and will shed the virus in their urine or saliva. On average, 30%-40% of childcare attendees who are 1-3 years of age are excreting CMV. In some studies, as many as 70% of children excrete the virus.^59^ Because CMV is so prevalent in the population and children shed the virus without symptoms, those with known CMV do not need to be excluded from entering the family-centered residential facility. Measures to prevent CMV transmission include frequent handwashing or use of alcohol-based hand sanitizer, avoiding contact with body fluids, and cleaning hands whenever exposed to body fluids. There is no vaccine available for CMV.

#### Recommendations

1. Allow individuals with current or past CMV infection to enter the family-centered residential facility
2. Instruct guests and visitors on good hand hygiene, especially after handling diapers and touching body fluids like saliva and nasal secretions
3. Remind guests and visitors to avoid sharing unwashed cups and utensils
4. Inform pregnant people about the risk of CMV infection and educate them about ways to prevent infection.

### Diarrheal illness with or without vomiting (bacterial or viral)

#### Background

Diarrhea may be due to infectious and noninfectious causes. Fever, vomiting, and loose stools (stools that take the shape of a container) that sometimes contain blood or mucus often accompany infectious diarrhea. Some viral and bacterial causes of diarrhea are associated with potentially severe illness, are highly contagious, and are transmitted by direct contact with infected feces or objects that have been contaminated with feces (e.g., diapers, clothing, changing tables, and toys).

Rotavirus and norovirus are two viruses that spread easily from person-to-person. Rotavirus diarrhea primarily affects infants. The incidence of rotavirus in infants has decreased since the reintroduction of the rotavirus vaccine in 2006, but children who are unvaccinated are at risk of this type of diarrhea.

Norovirus easily spreads among healthy individuals of all ages. Some people with norovirus have vomiting without diarrhea. There is no vaccine available for norovirus.

Follow these recommendations if any person develops new diarrhea or vomiting, including when the cause is unknown.

#### Recommendations

1. Restrict individuals with acute diarrhea with or without vomiting from entering the family-centered residential facility until they have been fever-free and diarrhea-free (without the use of fever-reducing or anti-diarrheal medications) for at least 24 hours. Do not require proof of having received antibiotic treatment to enter the facility. Some types of bacterial diarrhea require antibiotic treatment and some types do not.
2. Restrict individuals with the following types of bacterial diarrhea and individuals who do not have symptoms of illness but are known to be shedding these bacteria until they receive **written medical clearance** for entry:
  - Shiga toxin-producing *Escherichia coli* (also called *E. coli* O157:H7)
  - *Shigella*
  - *Salmonella* serotype Typhi (the cause of typhoid fever).
3. Restrict individuals with known norovirus or rotavirus infection from entering the facility (see **recommendation 1**). Both viruses spread easily from person-to-person and have a high risk of causing outbreaks. Younger children, especially those who are diapered, may shed high concentrations of these viruses in their stools.^60,61^ Environmental cleaning and disinfection is especially important when these viruses cause diarrhea. Use of a bleach-containing product or a diluted bleach solution should be used for cleaning when norovirus is suspected or diagnosed or when an outbreak of diarrheal illness occurs.
4. Restrict individuals who had diarrheal illness within the previous 2 weeks from food preparation and food handling in common areas.
5. Allow individuals who were exposed to a person who has diarrhea but who do not have symptoms of illness to enter the facility.
6. Educate guests and visitors about:
  - The importance of frequent hand hygiene
  - When hands are visibly soiled or they have potentially been in contact with surfaces with diarrheal-causing germs, they should wash them with soap and water for at least 15 seconds over using alcohol-based hand sanitizer.^62^
  - Appropriate, prompt removal and careful handling of contaminated items (e.g., diapers, clothing, and linens).
7. Refer any guest who develops diarrhea for medical evaluation. Until they are evaluated, require that they and their family members restrict themselves to their private room.
8. In severe or outbreak situations, notify the local health department and affiliated healthcare facility for guidance and potential recommendations for additional restrictions.

### Diphtheria

#### Background

Diphtheria is an infection caused by the bacteria *Corynebacterium diphtheriae.* This vaccine-preventable disease is rare in North America and most of Europe but continues to cause life-threatening disease in Africa, Latin America, Asia, the Middle East, and parts of Europe where vaccination coverage is suboptimal. Respiratory diphtheria can cause fever, a severe sore throat and swollen glands in the neck. Diphtheria can also cause a skin infection with a painful, open sore. Severe diphtheria is associated with blockage of the upper airway, myocarditis (severe inflammation of heart muscle), and nerve damage.^63^

#### Recommendations

1. Restrict individuals with confirmed or suspected diphtheria from the family-centered residential facility. Allow entry to the facility when they can provide **written medical clearance** from a licensed clinician or local public health department stating that they are no longer contagious (this includes written documentation of completion of the recommended course of antibiotics (usually at least 10 days of antibiotics, depending on specific disease) *and* 2 negative cultures proving eradication of bacteria.^63^
2. *Regardless of their immunization status*, restrict individuals exposed to a person with confirmed or suspected diphtheria who do not have symptoms of illness from the facility until they obtain **written medical clearance** from a licensed clinician or public health department, stating that they are not a risk for transmitting the infection.
3. Refer individuals who develop symptoms of diphtheria for medical evaluation. Instruct the individual and family members to leave the facility immediately. Contact the local public health department for further instruction. If the individual is diagnosed with diphtheria, notify the public health department and the program manager. The program manager should identify, notify, and instruct exposed guests to contact their primary care physician or licensed clinician and the public health department, which may recommend post-exposure testing and preventative treatment regardless of immunization status.

### Hepatitis A

#### Background

Hepatitis A is a virus that usually spreads through contact with infected persons and by eating or drinking contaminated food or water. In recent years, hepatitis A outbreaks have occurred among people who have eaten contaminated foods, had close contact with others who are infected, and among people who use drugs, are experiencing homelessness, and by men who have sex with men. The illness is characterized by fever, fatigue, jaundice (yellow skin), poor appetite, and nausea. It also rarely causes severe liver disease, usually in people with underlying chronic conditions.^64^

Individuals with acute hepatitis A infection are most infectious 1-2 weeks before they develop jaundice or other symptoms.^64^ High levels of the virus are excreted in the stool during those weeks, then the amount of virus decreases substantially one week after jaundice appears.

The hepatitis A vaccine is recommended for children and adolescents, for those at risk for severe disease from hepatitis A, and for those in settings that provide services to adults with risk factors for hepatitis A, including group homes.

#### Recommendations

1. Restrict individuals with hepatitis A infection from entering the family-centered residential facility until they receive **written medical clearance** that states at least one week has passed since the illness began and that the individual is no longer contagious.
2. Allow individuals who have been exposed to a person with hepatitis A to enter the facility, *only* if they are vaccinated against hepatitis A with 2 doses of vaccine. Outbreaks of hepatitis A can occur in unvaccinated households, and the incubation period can be prolonged (15-50 days).^64^
3. Educate all family members about the importance of frequent hand hygiene and appropriate, prompt removal and careful handling of contaminated items (e.g., diapers, clothing, and linens). When hands are visibly soiled or potentially have come in contact with stool, instruct them to wash their hands with soap and water, rather than alcohol-based hand sanitizer.
4. Refer individuals who develop symptoms of hepatitis A for medical evaluation. Instruct the individual and family members to leave the facility immediately. If the medical evaluation diagnoses the person with hepatitis A, notify the local public health department and program manager. The program manager should identify, notify, and instruct exposed guests to contact their physician or public health department. Exposed individuals may require post-exposure vaccination or immunoglobulin.^65^
5. In severe or outbreak situations, the local health department or affiliated medical facility may enforce additional restrictions.

### Hepatitis B, Hepatitis C, and Human Immunodeficiency Virus (HIV)

#### Background

##### Hepatitis B virus

Hepatitis B virus (HBV) can cause acute and chronic liver disease. Some patients may appear healthy, but others may be ill with nausea, vomiting, jaundice (yellowing of the skin), or severe liver disease.^66^ Chronic infections may lead to cirrhosis (scarring of the liver), liver failure, and cancer.^67^

HBV spreads by infected blood or body fluids. Transmission from an infected individual can occur by transfusion of blood that has not been screened for HBV, sharing or using non-sterile needles, syringes or glucose monitoring devices, needle stick injuries, and sexual contact with an infected person or household exposure to a person with chronic HBV. HBV can also be spread from mother to infant during pregnancy or at the time of birth.^66,67^ Transmission of HBV infection in childcare settings has been described but occurs rarely. Such transmission is most likely to occur through direct contact with blood from an infected person after an injury or from bites or scratches that break the skin.^24^ Vaccination provides long-term protection against hepatitis B virus infection. Hepatitis B vaccination is recommended for all infants in the United States; the first dose is given at the time of birth or before being discharged from the nursery.

##### Hepatitis C virus

Like HBV, hepatitis C virus (HCV) also causes acute and chronic liver disease. Chronic HCV infections may lead to liver failure and cancer.^68^ Individuals who are infected with HCV may feel well and not know they are infected.^68^

HCV spreads through infected blood or body fluids. The most common risk factors are sharing or using non-sterile needles, and male-to-male sexual contact. HCV can also be spread from mother to infant, and rarely, by household contact.^68^ The transmission risks of HCV infection in childcare settings are unknown, but are likely to be extremely low.^24^ Currently, there is no vaccine available to protect against HCV infection, but there are effective antiviral treatments.

###### Human immunodeficiency virus

Human immunodeficiency virus (HIV) causes an infection that attacks the immune system and has a variety of symptoms. In its advanced stage, it causes acquired immunodeficiency syndrome (AIDS), which is associated with increased risk for many life-threatening infections.^69^ Transmission of HIV only occurs by contact with blood or certain body fluids (e.g., semen, vaginal secretions, and human milk). Transmission does not occur by casual contact and has not been documented in settings such as school or childcare.^69^

Currently, there is no vaccine to protect against HIV infection, but there are effective medications that greatly reduce the risk of transmission from person-to-person.

#### Recommendations

1. Allow individuals who are infected with HBV, HCV, or HIV to enter the family-centered residential facility without restrictions, except for in the following situations:
  - Restrict entrance of an individual who has an HBV, HCV, or HIV infection *and* open sores on their skin that cannot be covered, or other conditions that might allow contact with their body fluids.
  - Consider specific restrictions, determined on an individual basis, if an infected person or their family member has behaviors (e.g., biting) that might put others at risk of contact with their blood or body fluids.^24,70,71^
2. Allow individuals who have been exposed to a person infected with HBV, HCV, or HIV to stay at the facility without restriction.
3. Educate families about the importance of appropriate, prompt disposal of items contaminated with blood and or body fluids (e.g., bandages, sanitary pads or tampons, and items that were used for injection of medication).^24,69^ Educate family members about the importance of good hand hygiene, appropriate glove use, and appropriate handling of blood and body fluids.^70,71^ Advise that they should not share personal items such as toothbrushes or razors.

### Herpes Simplex Virus (HSV) infections

#### Background

Herpes simplex viruses (HSV) cause a spectrum of clinical infections, including:

1. Cold sores or “fever blisters” (rashes made up of painful clusters of small, fluid-filled blisters)
2. Genital rashes or ulcers
3. Sores on the fingers or toes, called “herpetic whitlow”
4. A severe skin rash in individuals with eczema (called *eczema herpeticum*)
5. Primary HSV gingivostomatitis, which is common in young children and associated with many ulcers in and around the mouth, drooling, high fever, and swollen lymph nodes in the neck
6. Severe, life-threatening illness in young infants that can affect the brain, liver, and lungs. Newborns who are infected with HSV may or may not have a skin rash.
7. Encephalitis or meningitis in older children and adults.

Like varicella, the chickenpox, and shingles virus, HSV stays in the body after a person’s first infection. Reactivation of HSV-related skin rashes and cold sores is common. Cases of oral HSV may not be obvious and the virus may be present in saliva even in the absence of visible sores.^72^ The virus spreads though contact with infected sores or contaminated secretions. People with cold sores should avoid kissing or having their mouths come in contact with newborns or immune compromised individuals. There is no vaccine available for HSV.

#### Recommendations

1. Allow individuals who have isolated labial (lip) cold sores to enter the family-centered residential facility if they are capable of frequent hand hygiene and can follow instructions not to kiss others. If the person is young or incapable of hand hygiene, then restrict them from entrance until the cold sore is completely crusted and dry or restrict them to their private room.
2. Restrict individuals with active HSV affecting their skin or eyes from entering the facility until they have obtained **written medical clearance** from a licensed clinician. In general, the individual must be able to completely cover the rash with clothing, bandage(s), or other appropriate dressing. If the rash is unable to be completely covered, restrict entrance until the rash is completely crusted and dry. If permitting entry, restrict the individual to their private room until the rash is completely crusted and dry.
3. Restrict those with primary herpes gingivostomatitis (see **5.** above) from entering the facility if active sores and drooling are present. Once they have recovered from the illness, require **written medical clearance** from a licensed clinician that states that the patient is no longer contagious.
4. Allow individuals who have been exposed to a person who is infected with HSV who do not have symptoms of illness to stay at the facility without restriction.
5. Educate family members about the importance of frequent hand hygiene, avoidance of sharing personal items, and prompt removal and careful handling of potentially contaminated items (e.g., tissues, towels, and linens). Advise family to avoid direct, close contact with rashes to prevent person-to-person transmission.

### Lice

#### Background

Head lice infestation is common, particularly among school-aged children of all socioeconomic groups.^73,74^ Head lice are small wingless insects (about the size of a sesame seed) that prey on the blood of humans.^73^ They move by crawling. Head lice lay eggs (nits) in an individual’s scalp where they are incubated by body heat and remain attached firmly to the hair shaft until they hatch. Once hatched, the louse injects saliva into an individual’s scalp when it feeds, which causes the scalp to become irritated and itchy. An itchy scalp is the most common symptom of head lice infestation, but because scalp itching may not develop for several weeks after becoming infested, many individuals initially do not have symptoms. Occasionally, skin breakdown from scratching the affected site may result in a bacterial infection of the scalp.

Because head lice do not fly or hop,^57^ transmission mostly occurs from direct contact with the head of an infested individual, such as from sleeping in the same bed.^73,74^ Head lice can spread from by sharing a bed and/or personal items that have contact with the head (e.g., combs, brushes, pillows, and hats). Lice do not survive when away from the scalp for more than day. Of note, head lice do not transmit other infectious agents.^73^

#### Recommendations

1. Restrict individuals with suspected untreated head lice, and their household contacts, from entering the family-centered residential facility until they have received a medical evaluation.
  - If medical evaluation rules out head lice, allow the individual to enter the facility.
  - If medical evaluation confirms head lice, the individual should undergo treatment as recommended by a licensed clinician. Once treatment is complete, the individual may enter the facility.
  - Encourage bedmates of individuals with confirmed head lice to also be treated for having head lice, even if they do not have symptoms.
  - Instruct guests and family members to keep the affected individual’s personal items (e.g., combs, brushes, hats, and pillows) inside their private room.
2. Refer any guest who develops symptoms of head lice for medical evaluation. Restrict the symptomatic individual and their family members to their private room until they have been medically evaluated and confirmed to not have head lice. If they are confirmed to have head lice, follow the recommendations above.
3. Launder bed linens with water that is hotter than 130° F
4. Thoroughly clean the surfaces in the affected person’s room and vacuum the floor and furniture
5. Place any items that cannot be laundered (e.g., stuffed animals and pillows) in a plastic bag for 2 weeks prior to reuse.

### Measles

#### Background

Measles is a highly contagious viral infection. Measles is spread when an infected person coughs, sneezes or talks, sending droplets containing the virus into the air or onto a surface. Infection can occur if someone breathes the air containing the infectious droplets (the virus stays in the air for up to two hours) or touches a contaminated surfaces and then touches their own eyes, noses or mouths. Measles commonly presents with symptoms of fever, cough, nasal congestion, conjunctivitis (pink eye), and a red rash that begins on the face then spreads to the entire body.^75^ The measles virus (rubeola) can also cause ear infections, pneumonia, croup, hepatitis, encephalitis, and diarrhea.

Individuals are contagious from 4 days before the onset of rash through 4 days after the rash appears.^8,24,76^

Measles remains a leading cause of childhood disease and death globally. Outbreaks have occurred during the past several years due to importation of disease by travelers and infections in individuals who are immunized incompletely.^76^ The measles vaccine is safe and effective and is routinely recommended for all healthy children aged 12 months and older.

#### Recommendations

1. Restrict individuals from the family-centered residential facility who are suspected or confirmed to have measles. Allow entry into the facility when they are no longer contagious, usually 4 days after the appearance of the rash.^8^ Prior to entry, require **written medical clearance** from a licensed clinician or local public health department that states that they are no longer contagious.^24,76^
2. Restrict all individuals who have been exposed to a person with suspected or confirmed measles until they obtain **written medical clearance** from a licensed clinician or public health department that states that they are not a risk for transmitting the infection.
3. Refer individuals who develop symptoms of measles, and their family members, to leave the facility immediately and receive prompt medical evaluation. Notify the medical facility in advance that individuals are being referred for measles evaluation so that appropriate isolation precautions can be initiated and exposures to others are prevented.
4. Contact the referring medical facility and local public health department for further instruction and assistance with testing to confirm the measles diagnosis.
5. If medical evaluation confirms the individual has measles, notify the person’s primary care physician or licensed clinician and the program manager immediately, who will:
  - Identify and notify exposed guests and instruct them to immediately consult a licensed clinician or the public health department for possible post-exposure prophylaxis.
  - Non-immune individuals who have a normal immune system and are not pregnant should receive measles vaccine up to 72 hours after exposure.
  - High-risk individuals who have a compromised immune system may receive immune globulin up to 6 days after exposure to measles.

### Meningitis (bacterial or viral)

#### Background

##### Bacterial meningitis

Bacterial meningitis is a serious infection of the membranes that cover the brain and spinal cord. A person who has bacterial meningitis may have a fever, severe headache, vomiting, and neck stiffness. Bacterial meningitis can lead to hearing loss, brain damage, and learning disabilities.

Most types of bacterial meningitis do not spread easily from person-to-person, except two specific types that can be highly contagious:

1. *Haemophilus influenza* type b (Hib) meningitis
2. *Neisseria meningitidis* meningitis (also called “meningococcal meningitis”), which also can cause other types of serious infections, such as bloodstream infections.

These bacteria can spread from person-to-person by inhaling or having direct contact with an infected person’s respiratory tract secretions^77^ through coughing, kissing, and sneezing. People who have close contact with a person who is sick with Hib or meningococcal meningitis are at increased risk of contracting the disease. Exposed contacts are sometimes given preventative antibiotics to prevent them from becoming sick^77^. If a person has been exposed to someone with bacterial meningitis, they should contact their primary care physician or licensed clinician, or the local public health department to find out whether they should be treated with post-exposure antibiotics.

Hib infection is rare because individuals can receive a vaccine that protects against this infection. Although meningococcal vaccines are available, not all of them protect against meningitis by all meningococcal bacterial strains.

##### Viral meningitis

Individuals who have viral meningitis have symptoms of fever, headache, vomiting, and neck stiffness. Unlike bacterial meningitis, most types of viral meningitis are not as severe as bacterial meningitis and do not spread as easily from person-to-person. Enteroviruses are common causes of viral meningitis and are spread through direct contact with stool (diarrhea) and respiratory fluids (coughing, saliva), or by touching items contaminated with stool or respiratory secretions.^78^ Some types of viral meningitis are transmitted by mosquitoes (e.g., West Nile virus) and do not spread from person to person.

#### Recommendations

1. Allow individuals who were discharged from the hospital after treatment for bacterial meningitis to enter the family-centered residential facility. An individual with bacterial meningitis who has received 24 hours of antibiotic therapy and been discharged is no longer contagious.
2. Allow individuals who were exposed to a person with bacterial or viral meningitis who do not have symptoms of illness to enter the facility without restrictions and refer them to their primary care physician or licensed clinician or to the public health department to determine if they qualify for post-exposure antibiotics to prevent the disease.
3. Allow individuals who are prescribed post-exposure antibiotics to enter the facility. The antibiotics may be indicated for the exposed person’s own health and safety, not because they pose a risk to others.
4. If a guest develops symptoms of meningitis, immediately refer them for medical evaluation. Notify the program manager if the medical evaluation finds that the person has Hib or meningococcal meningitis. In such cases, the program manager should identify, notify, and instruct exposed guests to contact their primary care physician, licensed clinician or public health authorities immediately to find out if they should receive post-exposure antibiotics.
5. Restrict entrance to the facility to individuals who have been discharged from the hospital after treatment for viral meningitis until they are fever-free for 24 hours and diarrhea (if present) has resolved completely.

### Mpox (formerly known as monkeypox)

#### Background

Mpox (formerly known as monkeypox) is a viral illness endemic to West and Central Africa that in 2022 became a global outbreak with over 30,000 cases and more than 40 deaths in the United States. Cases of mpox in children were rare but a more recent outbreak in the Democratic Republic of the Congo has included children.^79^

Symptoms of mpox include fever, swollen lymph nodes, and flu-like symptoms like headache and malaise. A rash appears 1-4 days after the onset of fever. The rash begins as flat red or discolored areas that become small, raised bumps then vesicles and finally sores filled with pus. The rash often starts on the face or the mouth, and then moves to the arms and legs, including the palms and soles. Over time, the sores may develop a central dimple and ultimately form a crust. Mpox can look like other common childhood illness such as chickenpox or hand foot and mouth disease.

Mpox typically spreads by direct contact with the rash. Less commonly, it can spread by prolonged periods of face-to-face interaction because of exposure to saliva and upper respiratory secretions.^80^ People with mpox are infectious from the time symptoms start until the rash is fully healed. Mpox has been spread by contaminated linens, but in the recent outbreak, the risk of spread via contaminated surfaces was low.^81^

#### Recommendations

1. Restrict individuals with confirmed or suspected mpox infection (painful, undiagnosed rash) from entry into the family-centered residential facility.
2. Refer any individual who develops a rash suspected to be mpox for prompt medical evaluation. Restrict those individuals to a private room until evaluation is complete.
3. Contact local public health department if an individual in the facility is diagnosed with mpox.
4. Individuals exposed to mpox but who do not have symptoms of illness do not need to restricted from entry unless directed by the local public health department.^82^
5. Clean and disinfect all surfaces touched by someone who has been found to have an mpox infection. Disinfect per CDC recommendations using an EPA-registered hospital-grade disinfectant approved for emerging pathogens list Q, which can be found at www.epa.gov.^83^

### Multidrug-resistant organisms (MDROs)

#### Background

Bacteria that have become resistant to the common antibiotics are referred to as multidrug-resistant organisms (MDROs). Some people may have an MDRO living on their skin or in their bodies but they are not ill. This is called being “colonized” with an MDRO. People can be colonized with an MDRO intermittently or consistently.^84,85^

People with chronic medical conditions (e.g., cancer, kidney failure, and transplant recipients) are at highest risk of becoming colonized or infected with an MDRO. There may be special circumstances when a referring hospital has experienced high transmission rates of a specific MDRO; therefore, additional restrictions may be recommended by the affiliated medical facility’s IPC department. Vaccines are not available for the prevention of the MDROs described here.

##### Methicillin-resistant Staphylococcus aureus (MRSA)

Methicillin-resistant *Staphylococcus aureus* (MRSA) is a well-known MDRO. MRSA is a type of bacteria that is resistant to many antibiotics. Healthy individuals may be colonized with MRSA, and the bacteria may live inside their nostrils or on their skin.

MRSA infections frequently involve the skin, causing boils, or abscesses. MRSA also can cause very serious diseases, including pneumonia, infection of the bones and joints, and abscesses deep within the body.

MRSA can spread from person-to-person, especially when there is close contact with the infected fluid or pus that is draining.

##### Vancomycin-resistant enterococcus (VRE)

Vancomycin-resistant enterococcus (VRE) is a bacterium that lives in the intestines of some people and it can cause infection. VRE infections in children are rare, and they usually occur in people with chronic illnesses such as cancer.^86,87^

##### Gram negative bacteria

Multiple types of Gram-negative bacteria have developed resistance to multiple antibiotics. These highly resistant bacteria live in the intestines of some people and can cause infections. Studies have found many healthy people in the community are colonized with Gram-negative bacteria, but the bacteria usually are identified when a patient is hospitalized.

#### Recommendations

1. Allow individuals known to be “colonized” with an MDRO entry to the family-centered residential facility, as long as the person currently does not have symptoms of illness, is able to follow recommended hygienic practices, and is able to contain their body fluids (e.g., stool).
2. If a facility or its referring medical facility has experienced high transmission rates of a specific MDRO, additional restrictions may be considered. Consultation with local IPC specialists is recommended.
3. If the individual has a specific disease caused by an MDRO (e.g., skin infection or respiratory infection), please refer to the specific disease section for more guidance.
4. Allow individuals exposed to a person known to be colonized with an MDRO but who do not have symptoms of illness to enter the facility without restriction.
5. Educate all family members about the importance of frequent hand hygiene and appropriate removal and careful handling of contaminated items (e.g. diapers, clothing, and linens).

### Mumps

#### Background

Mumps is a viral infection characterized by the swelling of one or more salivary glands of the head and neck, usually the glands located on the face immediately in front of the earlobes. Rarely, it can cause complications affecting other organ systems of the body.^88^

Mumps spreads through contact with respiratory droplets and saliva. Individuals are most contagious several days before the appearance of swollen glands until 5 days after the onset of symptoms.^8,88^ Mumps can be prevented by vaccination.

#### Recommendations

1. Restrict individuals with mumps from the family-centered residential facility until at least 5 days from the appearance of swollen glands. Individuals must obtain **written medical clearance** from a licensed clinician before entering the facility.^8^
2. Restrict individuals who have been exposed to a person with mumps, even if they do not have symptoms of illness, from entry the facility until they obtain **written medical clearance** from a licensed clinician that states that they are not a risk for transmitting the infection.^24^ Exposed individuals can become ill with mumps 12-25 days after exposure.^88^
3. If someone in the facility develops symptoms of mumps, instruct the person and family members to leave the facility immediately and refer them for medical evaluation. If they are diagnosed with mumps, notify the local public health department and program manager immediately. The program manager should identify, notify, and instruct exposed guests to contact their primary care physician or licensed clinician, or the public health department.

### Pertussis

#### Background

Pertussis is an illness caused by *Bordetella pertussis* characterized by a prolonged cough.^89^ The typical incubation period for pertussis is 7-10 days, although rarely it can be as long as 21 days. Pertussis may start with a runny nose, sneezing, and a low-grade fever. Although during this initial phase a person’s cough may be mild, the high volume of the germ in their nose and throat increases the likelihood of transmission. During the next phase, people may begin to show classic signs of pertussis, including severe, episodic coughing with a “whooping” sound during their inhalation and forceful coughing followed by vomiting. Young infants who have pertussis may have difficulty feeding or may experience apnea (breath holding) during coughing spells. Pertussis infection can be life threatening or fatal in infants who have very small airways and are too young to be protected by immunization.

Immunity wanes 5-10 years after immunization, leaving adolescents and adults susceptible to getting pertussis.^90^ Although adolescents or adults may develop pertussis, they may have mild cough without the “whooping” sound on inhalation. The cough may sound like a “smoker’s cough,” bronchitis, or asthma.^91^

Outbreaks of pertussis have been reported in hospitals,^92^ schools,^93^ childcare centers,^94^ and other residential facilities.^95^ Pertussis spreads through large respiratory droplets that a person expels during coughing or sneezing. These droplets then contact another person’s mouth or eyes, or they are breathed in. Transmission also can occur after touching infectious droplets on a surface or object. Patients without classic or severe symptoms may transmit pertussis.^96^ Typically, infected people typically are infectious for 3 weeks if they do not get treated with appropriate antibiotics.

Vaccines are available to prevent pertussis in children and adults. A single dose of tetanus toxoid-reduced diphtheria toxoid and acellular pertussis (Tdap) vaccine is recommended for all adults who have close contact with infants younger than 12 months of age.^97^

#### Recommendations

1. Restrict individuals with pertussis from the family-centered residential facility until they have completed treatment and obtained **written medical clearance** from a licensed clinician. A person who has pertussis is contagious until they have completed 5 days of appropriate antibiotic therapy.^89^ Facilities do not need to restrict individuals who have received effective treatment but are still coughing. Cough may persist for many weeks after the illness.
2. During the initial routine screening process upon facility entry, screen family members and other close contacts of children who have respiratory symptoms. Infants and young children commonly acquire pertussis from a family member who has not yet sought medical care for their illness.^98^
3. Restrict individuals who have been exposed to pertussis from the facility, even if they do not have symptoms of illness, until active infection has been ruled out and preventative antibiotics have been started.^89^ Preventative antibiotics are indicated even if exposed people are up to date on immunization. **Written medical clearance** from a licensed clinician is required for a person who has been exposed to pertussis to be allowed to enter the facility.
4. Individuals exposed to pertussis who refuse or are unable to take their prescribed antibiotics should be restricted from the facility for 21 days after last pertussis exposure, even if they do not have symptoms of illness. Require **written medical clearance** from a licensed clinician before allowing entry.
5. If a guest is suspected to have pertussis, notify the program manager immediately and refer them to get a medical evaluation by their primary care physician or a licensed clinician. The program manager should notify the local health department to help identify and notify exposed guests.^92^

### Respiratory infections

#### Background

People can become ill with upper tract or lower tract respiratory infections. Upper tract respiratory infections include “the common cold,” sinusitis, and croup. Lower tract respiratory infections include pneumonia and bronchiolitis. Lower tract respiratory infections are associated with substantial morbidity and mortality. The risk of a person having a severe lower respiratory infection depends on a person’s age, underlying conditions, and the organism that is causing the infection.

The viruses adenovirus, rhinovirus, respiratory syncytial virus (RSV), human metapneumovirus, influenza viruses, parainfluenza viruses, and the coronaviruses most commonly cause upper respiratory tract infections.

Viruses or bacteria can cause lower respiratory tract infections. In infants and severely immune compromised individuals, these viruses can cause severe lower tract respiratory disease. In severely immune compromised individuals and people with underlying conditions (prematurity, chronic lung disease, heart disease, etc.), RSV pneumonia carries high morbidity and mortality.^99^ In infants, RSV bronchiolitis is the most common cause of hospitalization for respiratory disease.^100^ RSV can be associated with large outbreaks.^101^

In children and adults, bacteria such as *Streptococcus pneumoniae* and atypical bacteria, such as *Mycoplasma pneumoniae and Legionella,* cause pneumonia.

Most respiratory viruses spread through respiratory droplets expelled during coughing and sneezing. For certain respiratory viruses, such as RSV, a person also can get sick from direct contact with infected respiratory secretions or objects that have been contaminated with infectious droplets.

Respiratory infections caused by bacteria spread by droplets expelled by the person who is sick before they have received 24 hours of effective antibiotics.

It is important to be aware of respiratory viruses that may become epidemics or pandemics. Local health departments often send health alerts when there is a community at risk for spread of a dangerous virus. In 2009, the influenza virus H1N1 became a worldwide pandemic. In late 2019, the coronavirus SARS-CoV-2, which causes COVID-19, quickly spread across the globe becoming a multiyear pandemic (see Supplementary Material, Appendix C, **COVID-19** for additional information).

Vaccines are available that prevent disease due to influenza, RSV, and SARS-CoV-2. Individuals who are 6 months of age and older should receive an influenza vaccine annually. An RSV vaccine, approved by the FDA in 2023, is recommended for pregnant people to protect their newborns.^102^ New RSV vaccines have been approved for individuals 60 years and older.^103^ Infants who were born prematurely and those who are high risk for infection may receive a monthly dose of RSV antibody (palivizumab) during the RSV season. Another RSV antibody treatment (nirsevimab) can protect young or high-risk infants from severe RSV disease with only one injection.^104^

#### Recommendations

1. Restrict individuals with *viral* respiratory illnesses from entering the family-centered residential facility until they have been fever-free for 24 hours without taking fever-reducing medicine.
2. After they no longer have a fever, allow entry to the facility but require that guests restrict themselves to their private rooms until all their symptoms (e.g., runny nose, cough, and congestion) resolve. See **COVID-19** for specific information about management of individuals diagnosed with COVID-19.
3. Restrict individuals diagnosed with *bacterial* pneumonia from entering the facility until they have received 24 hours of effective antibiotics, and they have been fever-free for 24 hours without taking fever reducing medicine. If their infection is from *Legionella*, allow entry because *Legionella* pneumonia does not spread from person-to-person. See **Pertussis** and/or **COVID-19** for specific information about management of individuals diagnosed with these germs.
4. Allow individuals who have been exposed to a person with a respiratory illness and who do not have symptoms of illness to enter the facility. See **Pertussis** and/or **COVID-19** for specific information about management of individuals exposed to these germs.
5. Educate all family members about the importance of cough etiquette, frequent hand hygiene, and appropriate removal and careful handling of contaminated items (e.g. tissues, clothing, linens, and toys).
6. As the influenza season approaches and once the influenza vaccine becomes available, remind families of the importance of getting vaccinated every year. If It would be especially helpful for families to be directed to places where they can obtain vaccine. See **COVID-19** for specific information about the COVID-19 vaccine.

### Ringworm

#### Background

Ringworm or “tinea” is a type of skin infection caused by several different species of fungus. A ringworm infection can occur on the scalp (tinea capitis), the body (tinea corporis), the hands (tinea manuum), the feet (tinea pedis or “athlete’s foot”), the nails (tinea unguium), the groin or inner thighs (tinea cruris of “jock itch”), and the beard area (tinea barbae). Typical symptoms include red, itchy skin, a ring-shaped rash, and hair loss.

Ringworm spreads by contact with personal items from someone who^24^ has a ringworm infection, such as towels, clothing, or hair care items, or surfaces that are contaminated with the fungus. It also can spread by contact with an infected dog or cat.

Treatment for ringworm depends on where the infection is on the body. Over-the-counter topical medication can treat infections on the skin, but a prescription medication taken by mouth is used to treat infections on the scalp. There are rare reports of infections that are resistant to antifungal medicines.^105^

#### Recommendations

1. Allow entry of individuals who have ringworm infection once they have begun treatment for the infection. A rash should not be not used as a reason for excluding someone who has begun treatment, as a ringworm rash may persist for several weeks.^24^
2. Refer guests who develop a rash while staying at the facility for medical evaluation by their primary care physician or licensed clinician.

### Scabies

#### Background

Scabies is an itchy skin infestation caused by the mite *Sarcoptes scabiei var. hominis.* Outbreaks of scabies, especially crusted scabies, have occurred in hospitals,^106^ nursing homes,^107^ and other residential facilities, sometimes lasting for several months.

Common symptoms of scabies include an itchy, bumpy rash between the fingers and toes, around the wrists and the elbows, under the breasts, and in the genital area.^108^ A rash also may occur on the head, neck, palms, and soles in children.

Crusted or Norwegian scabies is a severe form of scabies that primarily affects the elderly and those who are immune compromised. Individuals with crusted scabies are very infectious because of the large number of mites present.

Scabies usually spreads by prolonged skin-to-skin contact with a person who is infected. Occasionally, contact with contaminated items such as clothing or linen can result in transmission.

#### Recommendations

1. Restrict individuals who have untreated scabies from entering the family-centered residential facility.
2. Allow entry to the facility once an individual’s treatment has been completed. Treatment usually can be done overnight. Itching may persist for several weeks after scabies treatment and should not be used as a reason for exclusion.
3. Refer all family members who were exposed to the person infested with scabies, even if they do not have symptoms, for medical evaluation by their primary care physician or licensed clinician. Typically, individuals who had prolonged skin-to-skin contact with a person infested with non-crusted scabies receive treatment.^109^ A facility does not need to exclude people who have been exposed to someone with scabies unless they have a rash.
4. Wash and dry all clothes and linens used by the affected person in hot water and dry with a high heat cycle to kill mites and prevent reinfestation. Temperatures more than 50°C or 122°F for 10 minutes will kill mites and eggs. Place any items that cannot be laundered (e.g., stuffed animals and pillows) in a plastic bag for at least 72 hours or up to one week.
5. Rooms occupied by guests who were subsequently diagnosed with scabies do not require the application of pesticides. Thoroughly clean and vacuum furniture and carpeting in a room occupied by a person with scabies to remove skin crusts and scales that may contain mites.
6. Crusted scabies is very contagious. A single case in the facility should prompt notification of the program manager and the local public health department to assist with investigation for additional cases, referrals for medical treatment, and control measures for cleaning clothing, bedding, and furniture.

### Skin and soft tissue infections (including abscess, cellulitis, and mastitis)

#### Background

Infections of the skin and soft tissues are caused most frequently by *Staphylococcus aureus* (also referred to as “Staph”) or *Streptococcus pyogenes* (also referred to as “Strep” or Group A strep; refer to the **Streptococcal infections** section), and other less common bacteria. Staph infections may be caused by staph strains that are very susceptible to antibiotics or by MRSA (see MRSA section above). Sometimes the specific bacteria can indicate the type of skin infection (e.g., impetigo, cellulitis, and abscess), the events leading to the infection (e.g., unknown, trauma, and animal bite), and the individual’s underlying risk factors (e.g., previous history of skin infections and immune compromise).^110^

Many skin infections are easily treated with oral antibiotics. Some skin infections, especially those caused by Staph, may require a minor surgical procedure to drain pus from an abscess (also called a boil), if present. If the infection is severe, it may require hospital admission for intravenous (IV) antibiotics.^110^

The ways these bacteria spread depends on the type of skin infection and the specific bacteria. Staph, including MRSA, transmit easily through direct contact with infected fluids, such as pus and through respiratory secretions from a person with pneumonia (see **MRSA** section).^110^ People can minimize the risk of transmission by avoiding direct contact with infected fluids, careful hand hygiene and using dressings to cover skin infections with draining fluid and changing dressings frequently.^111^

Individuals also can be colonized with these bacteria, meaning the bacteria live in their nose, throat, or on their skin, but they do not have symptoms of illness.^111^

#### Recommendations

1. If group A streptococcus (Strep) is the cause of the individual’s skin infection, refer to **Streptococcal infections** section.
2. Restrict individuals from entering the family-centered residential facility with skin and soft tissue infections if they have active drainage from the infection site. After an individual has surgical drainage of an infected abscess (boil), the facility may allow entry if the affected skin can be kept completely covered at all times with a clean and dry dressing.
3. Allow individuals who have been exposed to someone with a skin infection who do not have symptoms of illness to enter the facility.
4. Refer individuals who develop a skin infection while staying at the facility for medical evaluation. Until evaluated, restrict the individual to their private room.
5. Educate guests and family members about the importance of frequent hand hygiene and careful handling of contaminated items (e.g., used bandages, clothing, and linens) with appropriate, prompt cleaning or disposal.

### Streptococcal infections (including strep throat)

#### Background

The most common infection caused by group A streptococcus (GAS) is pharyngitis, commonly called strep throat. Symptoms include fever, sore throat, and enlarged lymph nodes in the neck. Scarlet fever can occur along with strep throat, and it is characterized by a red, sandpaper-like rash.^112^ GAS can cause other infections including skin and soft tissue infections, pneumonia, musculoskeletal infections, bloodstream infection, and sepsis. Sepsis is the body’s overactive and toxic response to infection. Sepsis can be life threatening.

GAS can spread through contact with the respiratory secretions of a person who is infected.^112^ A person is considered contagious until they have been treated with appropriate antibiotics for 24 hours.^24^ The is no vaccine available to prevent GAS infections.

#### Recommendations

1. Restrict individuals who have active, untreated GAS infections from entry to the family-centered residential facility.
2. Allow entry once they have completed at least 24 hours of antibiotic treatment and have been fever-free for 24 hours without the use of fever-reducing medication.^8,24^
3. Allow individuals who were exposed to someone with a documented case of GAS infection to enter the facility if they do not have symptoms of illness.
4. Refer for medical evaluation individuals who were exposed to GAS and develop symptoms for testing and treatment if the test is positive. Apply the previous steps to their reentry to the facility.^24^

### Tuberculosis

#### Background

Tuberculosis (TB) is a disease caused by infection of the lungs and other organ systems with *Mycobacterium tuberculosis* bacteria. TB is transmitted by small airborne respiratory droplets that may remain suspended in the air and travel long distances. It usually spreads only through air and not by contact with surfaces or objects.^113^

Individuals with active TB should be considered contagious until they are treated with antituberculosis medications. They will receive follow-up testing to prove that they are no longer contagious. Those who are most at risk of getting TB have shared the same air space for a prolonged period in an enclosed environment with a person who has an active TB infection.^113^

A person can have latent TB (LTBI) after infection with *M. tuberculosis.* This occurs when the bacteria remain in the body but do not cause disease. A person with LTBI and no symptoms is not contagious.^113^ Sometimes the bacteria cause active disease in a person with untreated LTBI. This can occur at any time after their infection, but most commonly it happens 1-6 months following the infection. It is characterized by fever, chills, night sweats, weight loss, and cough.^114^ There is no vaccine available in the United States to prevent TB infection.

#### Recommendations

1. Restrict individuals with active tuberculosis from entry to the family-centered residential facility until they have **written medical clearance** from the treating licensed clinician or local public health department that they are considered non-infectious. Verbal medical clearance by direct telephone consultation with a public health official is also acceptable.
2. Allow individuals with LTBI infection to enter the facility.
3. Restrict all family members who were exposed to a person with active TB from entering the facility until they have **written medical clearance** from the treating licensed clinician or the local public health department that they are considered non-infectious. Verbal medical clearance by direct telephone consultation with a health department official is also acceptable.
4. Ask any guest with a new diagnosis of active TB and their family members to leave the facility immediately. Require **written medical clearance** for readmission to the facility per the instructions above. Based upon consultation with the local public health department, the program manager may need to identify and notify all exposed guests and instruct them to seek medical attention.^24^

### Varicella-zoster virus (chickenpox and shingles)

#### Background

The varicella-zoster virus is the cause of chickenpox and shingles. It is highly contagious.

##### Chickenpox

Chickenpox is characterized by fever and an itchy body rash of fluid-filled blisters on a red base (“dew drops on a rose petal”). The rash is typically scattered over the body, including the scalp, and eventually forms scabs. The severity of disease increases with age and is most severe in those with weakened immune systems. Chickenpox may also lead to pneumonia, brain inflammation (encephalitis), or bacterial infection. Routine vaccination against varicella reduces the risk of acquiring chickenpox.

A person with chickenpox is highly contagious^115,116^ starting from 2 days before the first appearance of the rash until all the blisters have completely dried and crusted.^117^ The virus is transmitted by respiratory secretions and direct contact with the blisters. At least one hospital outbreak involving a family-centered residential facility has been reported.^2^

##### Shingles

After a person recovers from chickenpox, the varicella-zoster virus remains dormant in their body. Shingles (also called herpes zoster) is an illness that occurs when the latent varicella-zoster virus becomes active again. A person with shingles has clusters of small, painful blisters. Sometime people can have pain without blisters or before the blisters occur. Shingles can develop anytime but is most common among those over 60 years of age and when a person is immune compromised. Pain may persist after the shingles blisters have healed.

A person with shingles is contagious until all their blisters have completely dried and crusted. The virus spreads during shingles through direct contact with the skin or contaminated items. In immune compromised patients, the virus also may be present in respiratory droplets. Although person-to-person transmission has been described in the hospital^118^ and adult long-term care facility^119^ settings, if an individual has a healthy immune system and can cover their localized rashes, transmission is unlikely to occur.

#### Recommendations

##### Chickenpox

1. Restrict individuals with suspected or active chickenpox from entrance to the family-centered residential facility.
2. Once the rash has crusted and the patient does not have a fever, they may be allowed to enter the facility. Prior to entry, require **written medical clearance** from a licensed clinician that confirms that the rash has completely crusted and the person is no longer contagious.
3. Supplementary Material Table A3 defines “significant exposures to chickenpox.”^117^ Restrict individuals with exposures to chickenpox until they meet the criteria in Supplementary Material, Table A4. Prior to entry, require written medical clearance from a licensed clinician that documents the reason(s) the exposed individual is not contagious (see Supplementary Material, Figure A1). To be considered not contagious, a person must meet 1 of 4 of these criteria (see Supplementary Material, Table A4)^117^:
  1. The person has a past history of having had chickenpox or shingles (diagnosed by a healthcare professional)
  2. The person has documentation of 2 doses of varicella-containing vaccine, separated by at least 3 months
  3. The person has documented varicella antibodies (also known as a positive varicella IgG blood test)
  4. It has been at least 21 days since the person’s last significant exposure to the person with chickenpox (if the exposed person received post-exposure prophylaxis, such as intravenous immune globulin (IVIG) or varicella zoster immune globulin, it must be 28 days rather than 21 since their last exposure).
4. Request that individuals with suspected chickenpox leave the facility immediately and seek medical evaluation. Require **written medical clearance** to return.
5. Contact the referring medical facility and local public health department for further instruction and assistance with testing to confirm the chickenpox diagnosis.
6. Individuals with chickenpox are considered contagious 2 days before the first appearance of a rash.^117^ If an individual diagnosed with active chickenpox entered the facility any time during the 2 days *before* the rash appeared, notify the program manager immediately, who then must identify and notify exposed guests and instruct them to consult their primary care physician or licensed clinician immediately to determine need for post-exposure prophylaxis. Post-exposure prophylaxis must be administered within 10 days of exposure to varicella.^120^

##### Shingles

1. Allow individuals with an active case of shingles to enter the family-centered residential facility only if they have **written medical clearance** documenting that these criteria are met:
  - The blisters are localized on one side of the body and can be completely covered by a dressing and/or clothing
  - The person has a normal immune system
  - The person is not taking medications that can weaken their immune system
  - The person is capable of frequent hand hygiene and understands the importance of caring for the blisters and the dressings to contain them.
2. If the person with shingles cannot meet all criteria above (see **a-d**), restrict them from the facility.
3. If the person with shingles meets criteria to allow entry, restrict the individual to a private room until the blisters have completely crusted.
4. Educate guests and visitors of the importance of frequent hand hygiene and appropriate disposal of potentially contaminated items (e.g., dressings, clothing, and linens).
5. People who are not immune to varicella can get chickenpox if directly exposed to shingles blisters. Interview individuals recently exposed to a person with shingles about the level of exposure to the blisters. If the person’s exposure was significant (see Supplementary Material, Table A3), then require written medical clearance prior to entry to the facility (see Supplementary Material, Table A1). The documentation should include the reason the exposed individual is not contagious by meeting 1 of 4 of these criteria (see Supplementary Material, Table A4)
  - The person has a past history of having had chickenpox or shingles (diagnosed by a licensed clinician)
  - The person has documentation of 2 doses of varicella-containing vaccine, separated by at least 3 months
  - The person has documented varicella antibodies (also known as a positive varicella IgG blood test)
  - It has been at least 21 days since the person’s last contact with the blisters (if the exposed person received post-exposure prophylaxis [intravenous immune globulin (IVIG)] or varicella zoster immune globulin, it must be 28 days since last exposure).^120^
6. Request that individuals suspected of having shingles leave the facility immediately and refer them for medical evaluation.
7. If medical evaluation rules out chickenpox and diagnoses the person with shingles, then refer to the above recommendations for managing a person with an active case of shingles.

## Supporting information

Guzman-Cottrill et al. supplementary material 1 Guzman-Cottrill et al. supplementary material

Guzman-Cottrill et al. supplementary material 2 Guzman-Cottrill et al. supplementary material

Guzman-Cottrill et al. supplementary material 3 Guzman-Cottrill et al. supplementary material

## Supplementary material

The supplementary material for this article can be found at https://doi.org/10.1017/ice.2024.124.
