# Supplementary material for: SHEA practice update: infection prevention and control (IPC) in residential facilities for pediatric patients and their families
Source: Infect Control Hosp Epidemiol. 2024 Nov 14;46(1):3–26. doi: 10.1017/ice.2024.124 (PMC11717477; doi:10.1017/ice.2024.124)
Supplement: Guzman-Cottrill et al. supplementary material 2 — Guzman-Cottrill et al. supplementary material [file S0899823X24001247sup002.pdf]

Disease Exposure Questionnaire for Residents and Visitors of RMHC® Programs

To RMHC® Staff: Please administer this questionnaire to all residents and visitors upon admission to program facilities and if/when families or patients develop new infectious symptoms. All RMHC® program residents and visitors **must** notify RMHC® Chapter staff of all new symptoms **immediately**.

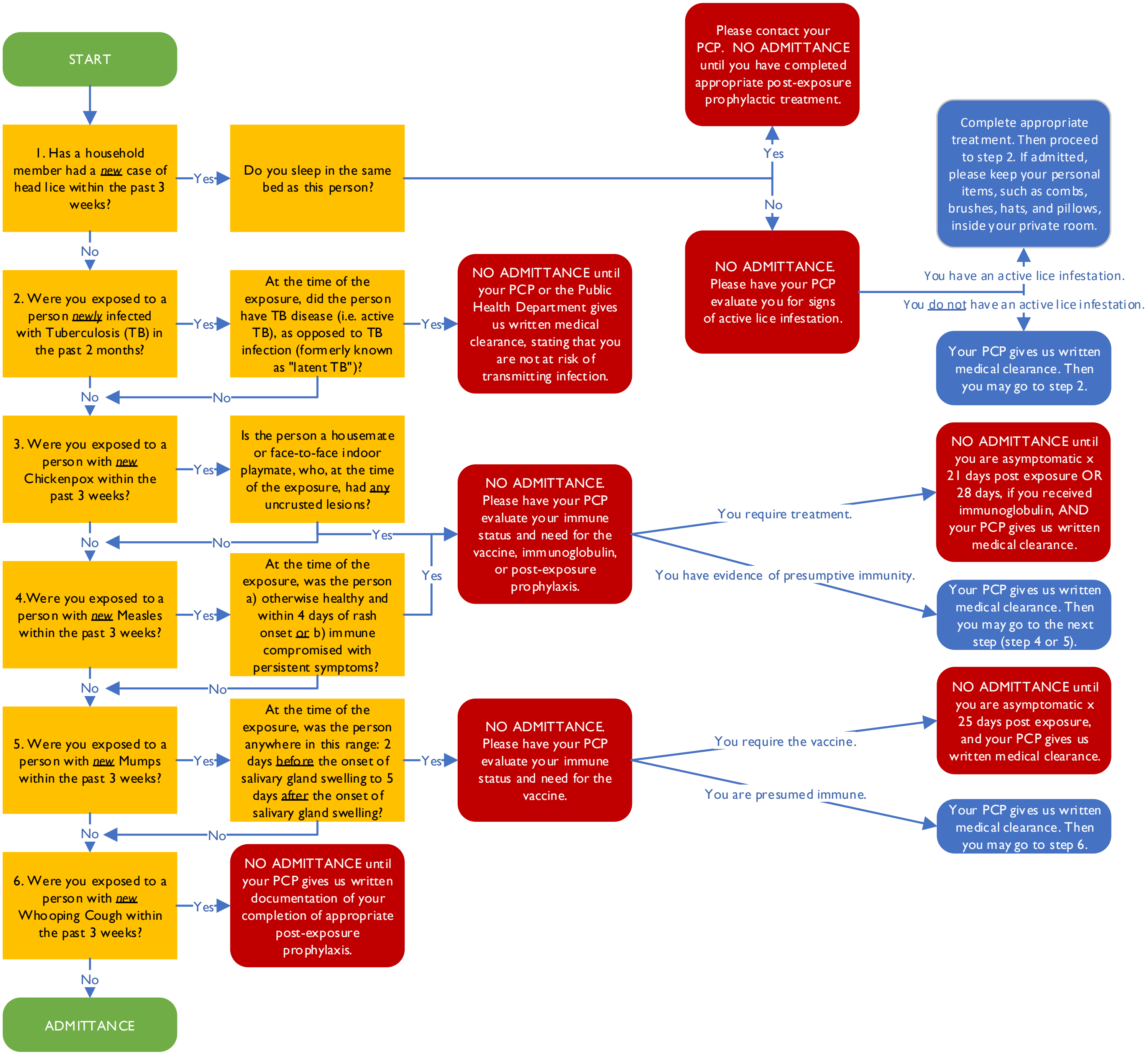

RMH = Ronald McDonald House
PCP = primary care provider
