# Supplementary material for: SHEA practice update: infection prevention and control (IPC) in residential facilities for pediatric patients and their families
Source: Infect Control Hosp Epidemiol. 2024 Nov 14;46(1):3–26. doi: 10.1017/ice.2024.124 (PMC11717477; doi:10.1017/ice.2024.124)
Supplement: Guzman-Cottrill et al. supplementary material 3 — Guzman-Cottrill et al. supplementary material [file S0899823X24001247sup003.docx]

SHEA Practice Update: Infection Prevnetion and Control in Residential Facilities for Pediatric Patients and their Families

*Executive Summary and Appendices*

Contents

[Executive Summary 2](#_Toc173168305)

[Scope 2](#_Toc173168306)

[Structure 3](#_Toc173168307)

[Core Principles of Infection Prevention and Control 4](#_Toc173168308)

[Specific Diseases and Pathogens 5](#_Toc173168309)

[Summary 5](#_Toc173168310)

[Demography and Definitions 5](#_Toc173168311)

[Figure 1. Four Steps to Food Safety 7](#_Toc173168312)

[Table 1. Recommended Internal Temperatures of Appropriately Cooked Food 7](#_Toc173168313)

[Table 2. Duration for Safe Storage in the Refrigerator and Freezer 9](#_Toc173168314)

[Appendix A 11](#_Toc173168315)

[Figure A1. Medical Clearance Form for Significant Infections 12](#_Toc173168316)

[Figure A2. Outbreak Response Checklist 13](#_Toc173168317)

[Table A3. Examples of Significant Varicella Exposures in a Family-Centered Residential Setting 15](#_Toc173168318)

[Chickenpox 15](#_Toc173168319)

[Zoster (shingles) 15](#_Toc173168320)

[Table A4. Criteria for Individual to be Considered “Non-Contagious” Despite Exposure to Varicella 15](#_Toc173168321)

[Appendix B 16](#_Toc173168322)

[Algorithm B1. Symptoms Questionnaire Algorithm (see PDF) 16](#_Toc173168323)

[Algorithm B2. Disease Exposure Questionnaire Algorithm (see PDF) 16](#_Toc173168324)

[Appendix C 17](#_Toc173168325)

[Table C1. Type and Duration of Precautions 18](#_Toc173168326)

[Components of Standard Precautions 18](#_Toc173168327)

[Definitions 18](#_Toc173168328)

[Abbreviations 19](#_Toc173168329)

[Type and Duration of Precautions Recommended for Selected Infections and Conditions 19](#_Toc173168330)

[Table C2. Frequently encountered special populations in family-centered residential facilities 30](#_Toc173168331)

[References 32](#_Toc173168332)

# Executive Summary

## Scope

The 2024 *Society for Healthcare Epidemiology of America (SHEA) Practice Update: Infection Prevention and Control in Residential Facilities for Pediatric Patients and Their Families* is the revised and updated infection prevention and control (IPC) document to address preventing transmission of infectious agents in “home away from home” residential settings, of which Ronald McDonald House Charities® (RMHC®) programs serve as a prototype. This 2024 practice update is meant to replace the original 2013 *Society for Healthcare Epidemiology of America (SHEA) Guideline: Infection Prevention and Control in Residential Facilities for Pediatric Patients and Their Families.*

These types of facilities provide support services including overnight lodging for children who are ill and injured and their families. Food preparation occurs in common areas and cleaning of rooms or apartments is performed by the occupants during their stay and before departure. Pediatric patients are frequent guests of the family-centered facilities while receiving or recovering from specialized medical therapy nearby. These facilities are located worldwide and vary in their physical structure and the predominant populations served.

Development of the 2013 guideline began after the presentation of IPC questions that two of the authors (JG, KR) had received from the local Ronald McDonald House (RMH) and Ronald McDonald Family Room (RMFR) programs to the SHEA Pediatric Leadership Council in October 2011. Infections can be reduced by following hospital IPC policies, but these policies are too stringent for a residential, family-centered facility. Following discussions with executive leaders of Ronald McDonald House Charities® (RMHC®), a guideline writing group was formed and a unique collaborative effort began with an initial needs assessment^1^ and establishment of a memorandum of understanding between SHEA and RMHC®. While RMHC® was involved in establishing this effort, the intention always has been to create a document that could be used by similar family-centered residential facilities.

If a family-centered residential facility is located within a medical facility, then the facility’s IPC-related policies and procedures will supersede the recommendations contained in this document. In most situations, a hospital policy or procedure will contain more detailed and structured instructions than this practice update provides.

This document differs from SHEA-endorsed guidelines and expert guidance documents:

1. The primary audience of this practice update is not healthcare personnel, but rather lay staff members and volunteers who are educating and monitoring visitors for illness or exposures in a program providing accommodation to children who are ill or injured and their families. Thus, the terminology used throughout the document was chosen to be understandable by individuals of varying educational backgrounds.
2. Peer-reviewed healthcare epidemiology literature related to this specific pediatric setting is sparse. The writing group developed recommendations by adapting currently available IPC evidence to this special setting. For topics where published evidence does not define best practices, this document provides practical recommendations. Ultimately, management decisions must be individualized for the specific circumstance and staff members are encouraged to rely on local expertise for complex problems. Many recommendations for IPC in daycare settings and outpatient clinics are applicable to RMH and RMFR programs, and similar facilities.
3. Literature searches were performed by the writing group and recommendations from guidelines published by the Centers for Disease Control and Prevention (CDC), SHEA, American Academy of Pediatrics (AAP), and the World Health Organization (WHO) when applicable. Recommendations were not assigned a “grade” based on quality of evidence and strength of recommendation because of the sparse evidence to support practices in this setting.

## Structure

This practice update contains several sections with background information to enhance the user’s understanding of the topics. Recommended practices for the facility staff are included immediately after the informational discussions. Sections included in the document are: 1) Introduction; 2) Background; 3) Infection Prevention and Equity; 4) Annual Staff Member and Volunteer Infection Prevention Education; 5) Core Principles of Infection Prevention and Control; 6) Food Safety; 7) Masking Guidance; 8) Animals; 9) Health Screening of Guests and Visitors of RMHC® Programs; 10) Staff Member and Volunteer Illness and Vaccination; 11) Breast Milk Storage and Maintenance of Breast Pumps; 12) Description of Special Populations often using the services of residential facilities and their vulnerabilities; 13) Protection of Highly Immune Compromised Patients from Exposure to Mold Spores; 14) Alphabetical list of specific diseases and pathogens (germs) with management recommendations; 15) Background literature; 16) Appendix A, with forms that may be used by staff; 17) Appendix B, with screening algorithms that may be used by staff; 18) Appendix C, which contains a summary table of infections with specific recommendations for a) exposed, healthy guests; b) guests with specified infections; c) restrictions needed within the facility; d) availability of special educational handouts for family members; and e) additional comments for each condition.

## Core Principles of Infection Prevention and Control

The core principles of IPC that form the foundation for recommended practices are ***Standard Precautions***, a set of practices aimed at preventing the spread of germs based on the principle that all blood, body fluids (e.g., material coughed or vomited, saliva, stool), non-intact skin, and mucous membranes ***may*** contain contagious germs. Therefore, these body fluids must be contained as much as possible.

The components of ***Standard Precautions*** and major recommendations in this document are as follows:

| ***Hand hygiene*** | Perform hand hygiene ***before*** preparing food or eating, ***before*** administering medication to a patient and ***after*** changing diapers, toileting or assisting others in toileting, or when hands are visibly dirty. |
| --- | --- |
| ***Glove use*** | Use gloves before contact with blood or body fluids; always perform hand hygiene after glove removal, and never wash and/or reuse the same gloves. |
| ***Respiratory hygiene/cough etiquette*** | Cover coughs and sneezes with a tissue, promptly throw away used tissues, and perform hand hygiene afterwards. Maintain three to six feet (one to two meters) from anyone coughing or sneezing. |
| ***Blood and body fluid precautions*** | Assume that all blood and body fluids may contain contagious germs (e.g., hepatitis viruses, human immunodeficiency virus). |
| ***Safe injection practices*** | Prevent accidental needlesticks and exposure to blood by handling all needles used for injection of medications carefully, not recapping used needles, using needles only once, and disposing used needles separately from all other trash into a rigid, puncture resistant “sharps” container. |
| ***Safe laundry management*** | Do not shake linens and clothing soiled with blood, vomitus, stool, etc. Separate these contaminated items in a plastic bag and wash separately in soap and hot water to prevent transmission of infectious agents. |
| ***Cleaning and disinfection of the environment*** | Create and follow routine housekeeping procedures in each residential facility. All items that require disinfection must first be cleaned with a detergent before using a disinfectant. Toileting and diapering areas must always be separate from food preparation areas. Surfaces and items in common areas and playrooms should be cleaned and disinfected frequently. Toys that are smooth and scrubbable are preferred, because they are easier to wipe down for cleaning and disinfection. |

## Specific Diseases and Pathogens

The purpose of this section is to provide practice recommendations for straightforward problems, (e.g., bed bugs, head lice, diarrhea), and education on how to recognize more complicated conditions, (e.g., chickenpox, whooping cough, measles, and tuberculosis exposures). While some conditions may be managed by the staff independently, others may require consultation with the referring hospital medical staff.

Vaccines help to maintain a healthy environment, reduce absenteeism, and prevent outbreaks. We recognize that it is not the primary responsibility of these facilities’ staff to assure appropriate vaccination status of its staff, volunteers, and guests. However, we strongly encourage these types of facilities provide families with information about vaccine access, especially for influenza and COVID-19 vaccines. Facilities also are encouraged to partner with local public health authorities to determine which immunizations are recommended and for whom. The following [World Health Organization (WHO) website](https://www.who.int/teams/immunization-vaccines-and-biologicals/policies/who-recommendations-for-routine-immunization---summary-tables) provides access to recommended routine immunizations for children, adults, and healthcare personnel around the world^2^. Similarly, CDC^3^ provides [immunization recommendations tables](https://www.cdc.gov/vaccines/schedules/index.html) for children, adults, and healthcare personnel.

## Summary

This practice update responds to the evolving changes in the delivery of healthcare to children worldwide, which frequently includes long distance travel for specialized medical treatment. Family-centered residential facilities located close to pediatric medical centers serve as bridges to returning home. The primary objective of this document is to improve the health of the dedicated staff members and volunteers, and the vulnerable patients and their families who utilize these family-centered facilities, which were developed to meet growing needs and improve the quality of life for children worldwide.

## Demography and Definitions

| Ronald McDonald House Charities® (RMHC®) program | a facility operated by a local RMHC® Chapter that provides overnight accommodations to families while their child receives medical care nearby. It may be freestanding or housed within a children’s hospital. |
| --- | --- |
| Ronald McDonald Family Room (RMFR) | a program within the hospital operated by a local RMHC® Chapter that provides day, and in many programs, overnight services for families while their children receive medical care. An example of an RMFR program is an area near a pediatric day hospital where children receive chemotherapy infusions. |
| Guest of an RMHC® Chapter or an RMHC® program | a family member or patient who utilizes RMH or RMFR programs’ services (including overnight stay) |
| Visitor of an RMHC® program | an individual who enters an RMHC® program but does not utilize their services, such as someone attending a RMH or RMFR program tour or visiting a guest of an RMHC® program |
| RMH program manager | an individual whose job responsibilities are to oversee the operations of the RMH or RMFR program and wellbeing of the families, staff, and volunteers under their supervision. |
| Staff member of an RMHC® program | a person employed by a local RMHC® Chapter |
| Volunteer for an RMHC® program | someone who contributes unpaid support to RMH or RMFR program daily operations |
| Licensed independent clinician | an individual permitted by law and by the healthcare facility to provide medical care without direction or supervision, within the scope of the state or local laws, individual’s license, and consistent with individually granted clinical privileges (e.g., physician, nurse practitioner, physician assistant, home healthcare provider). |

# Figure 1. Four Steps to Food Safety

**
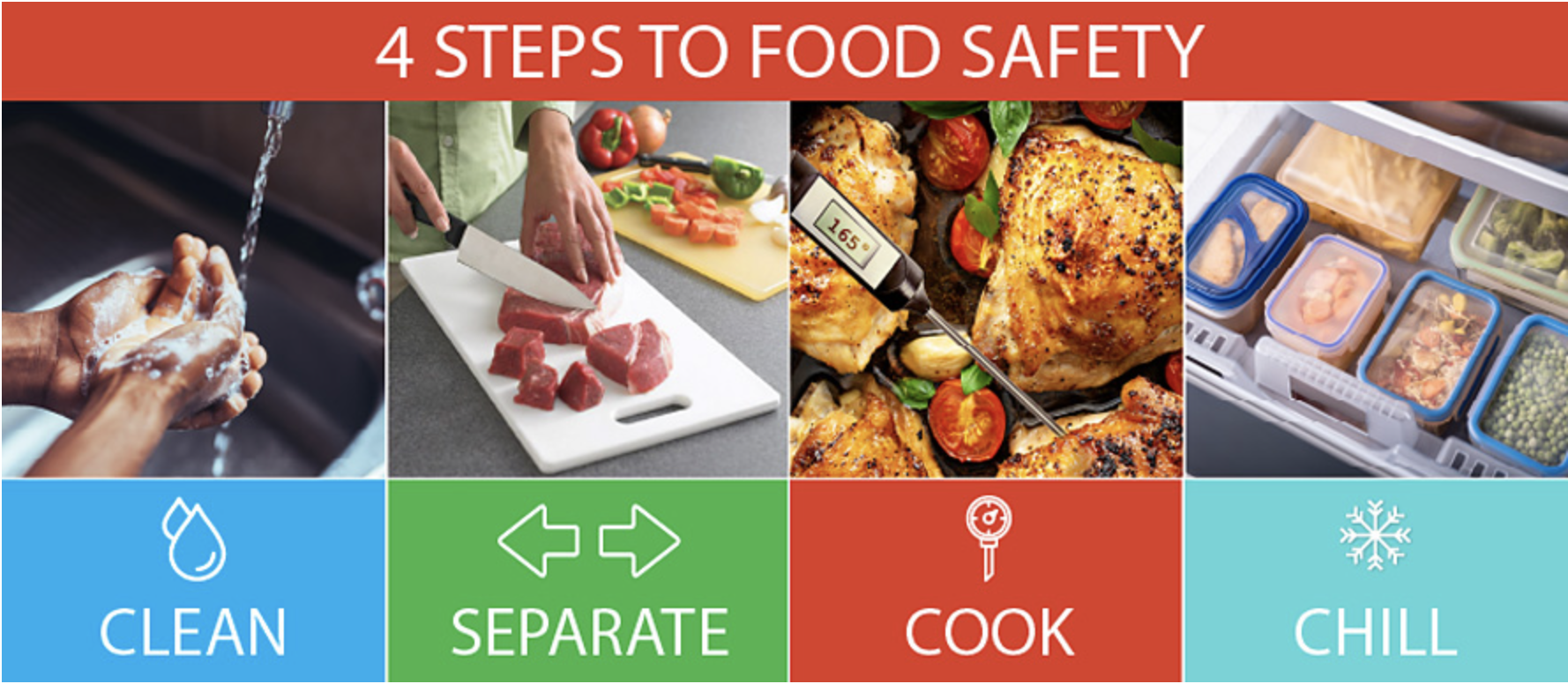
**

Figure 1. The US Centers for Disease Control and Prevention’s and Food and Drug Administration’s 4 simple rules for food safety.^4,5^

# Table 1. Recommended Internal Temperatures of Appropriately Cooked Food

| **Food** | **Type** | **Internal Temperature (^o^C/^o^F)** |
| --- | --- | --- |
| Beef, bison, veal, goat, and lamb | Steaks, roasts, and chops | 63^o^/145^o^  Rest time after cooking**: 3 minutes** |
|  | Ground meat and sausage | 71^o^/160^o^ |
| Casseroles | Meat and meatless | 74^o^/165^o^ |
| Chicken, Turkey, and other poultry | All: whole bird, breasts, legs, thighs, wings, ground poultry, giblets, sausage, and stuffing inside poultry | 74^o^/165^o^ |
| Eggs | Raw eggs | Cook until yolk and whites are firm |
|  | Egg dishes (such as frittata, quiche) | 71^o^/160^o^ |
|  | Casseroles containing meat and poultry | 74^o^/165^o^ |
| Ham | Raw ham | 63^o^/145^o^  Rest time after cooking**: 3 minutes** |
|  | Precooked ham | 74^o^/165^o^  Note: Reheat cooked hams packaged in USDA-inspected plants to 60^o^C/140^o^F |
| Leftovers | Any type | 74^o^/165^o^ |
| Pork | Steaks, roasts, and chops | 63^o^/145^o^  Rest time after cooking: 3 minutes |
|  | Ground meat and sausage | 71^o^/160^o^ |
| Rabbit and Venison | Wild and farm-raised | 71^o^/160^o^ |
| Seafood | Fish (whole or filet), such as salmon, tuna, tilapia, pollock, bass, cod, catfish, trout, etc. | 63^o^/145^o^ or cook until flesh is no longer translucent and separates easily with a fork |
|  | Shrimp, lobster, crab, and scallops | Cook until flesh is pearly or white and opaque |
|  | Clams, oysters, mussels | Cook until shells open during cooking |

Table 1. Recommended internal temperatures of appropriately cooked foods as per the US Centers for Disease Control and Prevention, Food and Drug Administration, and Department of Agriculture.^6^

# Table 2. Duration for Safe Storage in the Refrigerator and Freezer

| **Food** | **Type** | **Refrigerator**  **(4^o^C/40^o^F or below)** | **Freezer**  **(-18^o^C/0^o^F or below)** |
| --- | --- | --- | --- |
| Salad | Egg, chicken, ham, tuna, and macaroni salads | 3-4 days | Not applicable |
| Hot dogs | Opened package | 1 week | 1-2 months |
|  | Unopened package | 2 weeks | 1-2 months |
|  | Opened package or deli sliced | 3-5 days | 1-2 months |
|  | Unopened package | 2 weeks | 1-2 months |
| Bacon and sausage | Bacon | 1 week | 1 month |
|  | Sausage, raw from chicken, turkey, pork, or beef | 1-2 days | 1-2 months |
|  | Sausage, fully cooked from chicken, turkey, pork, or beef | 1 week | 1-2 months |
|  | Sausage, purchased frozen, after cooking | 3-4 days | 1-2 months |
| Hamburger, ground meats and poultry | Hamburger, ground beef, chicken, other poultry, veal, pork, lamb, and mixtures of them | 1-2 days | 3-4 months |
| Fresh beef, veal, lamb, and pork | Steaks, chops, and roasts | 3-5 days | 4-12 months |
| Ham | Fresh, uncured, uncooked | 3-5 days | 6 months |
|  | Fresh, uncured, cooked | 3-4 days | 3-4 months |
|  | Cured, uncooked | 5-7 days | 3-4 months |
|  | Fully cooked, vacuum-sealed at plant, unopened | 2 weeks | 1-2 months |
|  | Cooker, store-wrapped, whole | 1 week | 1-2 months |
|  | Cooked, store-wrapped, slices, half, or spiral cut | 3-5 days | 1-2 months |
|  | Country ham, cooked | 1 week | 1 month |
|  | Canned, labeled “Keep Refrigerated,” unopened | 6-9 months | Not applicable |
|  | Canned, shelf-stable, opened | 3-4 days | 1-2 months |
|  | Prosciutto, Parma or Serrano ham, dry Italian or Spanish type, cut | 2-3 months | 1 month |
| Fresh poultry | Chicken or turkey, whole | 1-2 days | 12 months |
|  | Chicken or turkey, pieces | 1-2 days | 9 months |
| Fin fish | Fatty fish (i.e. bluefish, catfish, mackerel, mullet, salmon, tuna, etc.) | 1-3 days | 2-3 months |
|  | Lean fish (i.e. cod, flounder, haddock, halibut, sole, etc.) | 1-3 days | 6-8 months |
|  | Lean fish (i.e. pollock, ocean perch, rockfish, sea trout, etc.) | 1-3 days | 4-8 months |
| Shellfish | Fresh crab meat or lobster | 2-4 days | 2-4 months |
|  | Live crab or lobster | 1 day | Not applicable |
|  | Live clams, mussels, oysters, and scallops | 5-10 days | Not applicable |
|  | Shrimp, crayfish | 3-5 days | 6-18 months |
|  | Shucked clams, mussels, oysters, and scallops | 3-10 days | 3-4 months |
|  | Squid | 1-3 days | 6-18 months |
| Eggs | Raw eggs in shell | 3-5 weeks | Not applicable |
|  | Raw egg whites and/or yolks, out of the shell | 2-4 days | 12 months |
|  | Hard-cooked eggs | 1 week | Not applicable |
|  | Egg substitutes, liquid, unopened | 1 week | Not applicable |
|  | Egg substitutes, liquid, opened | 3 days | Not applicable |
|  | Pies, after baking | 3-4 days | 1-2 months |
|  | Quiches, after baking | 3-5 days | 2-3 months |
| Leftovers | Cooked meat or poultry | 3-4 days | 2-6 months |

Table 2. Recommended timeframes of refrigerated and frozen foods before they should be discarded per the US Centers for Disease Control and Prevention, Food and Drug Administration, and Department of Agriculture.^7^

# Appendix A

## Figure A1. Medical Clearance Form for Significant Infections

**Medical Clearance Form for Disease-Specific Infection Exposures**

***This form must be completed by a physician or public health representative who is currently*** ***involved in the child’s health care.***

| Parent/Legal Guardian Name (PRINT) |  |
| --- | --- |
| Pediatric Patient’s Name (PRINT) |  |

| **Pediatric Patient’s Diagnosis** (circle): | | |
| --- | --- | --- |
| Chickenpox (varicella) | Measles | Tuberculosis |
| E coli colitis/diarrhea/HUS | Mumps | Whooping Cough (pertussis) |
| Hepatitis A | Salmonella colitis/diarrhea | Other: |
| Herpes Infection (HSV-1 or HSV-2) | Shigella colitis/diarrhea |  |

***I am involved in the child’s care (named above) and have determined the exposed family members are NOT contagious to other residential facility guests.***

Please mark as appropriate below:

- Family members have a history of completed immunization *or* natural disease immunity against this specific disease.
- Family members have completed a full course of appropriate antibiotic exposure prophylaxis against this specific disease.
- Family members have been evaluated and cleared by the public health department.
- Family members are currently not ill and are not considered a risk since they are currently healthy.

**Physician/Public Health Representative:**

| PRINT NAME |  | Today’s Date: |
| --- | --- | --- |
| SIGNATURE |  | Contact Telephone Number: |

## Figure A2. Outbreak Response Checklist

***What is an outbreak?***

An outbreak is a sudden, unexpected increase in the number of cases of a disease. This could be one case of a rare infection, or multiple cases of an infection linked in time. Outbreaks are rare but can grow quickly. Prompt recognition and action are vital to ensure the health and safety of those in a facility experiencing a disease outbreak. Staff members of an RMH or RMFR program may recognize an outbreak in guests or staff members. Alternately, they may be contacted by local public health authorities or a hospital partner with concerns about an outbreak. The checklist below can be used as a framework for how outbreaks are managed.

***Use this checklist to guide decision-making if an outbreak occurs:***

|  | **Action** | **Description** | **Example (norovirus)** |
| --- | --- | --- | --- |
| **1** | **Recognize** | When guests of RMH programs become ill, there should be a concerted effort to be on the lookout for additional people with similar illness. When an outbreak (multiple guests or staff members with same illness over a short period of time) is suspected, prompt response is initiated. | Two staff members and four guests develop vomiting and diarrhea 24 hours after a holiday party. One guest, a child with cancer, is treated at the emergency department for dehydration and tests positive for norovirus. An outbreak is suspected. According the CDC, a norovirus outbreak is defined as the occurrence of two or mor similar illnesses resulting from a common exposure that is either suspected or confirmed to be caused by norovirus.^8^ |
| **2** | **Identify** | Outbreak response requires identifying those who are ill and those who may be exposed to illness. Encourage guests to self-report illnesses to keep others safe. When an outbreak is suspected, create a [line list](https://www.cdc.gov/urdo/downloads/linelisttemplate.pdf) that includes at least the age of each ill person, their symptoms, and the date of onset of symptoms. Symptom screening during outbreaks may be an effective method to identify these guests.^9^ | A list of sick guests and staff members is created using the line list tool. A sign is posted at the welcome desk and the community room asking guests to notify the RMHC® program supervisor if they develop vomiting or diarrhea. Staff members are asked during shift change and by email to report gastrointestinal symptoms. |
| 3 | Collaborate | Reach out to community partners as soon as an outbreak is suspected. These could include local public health authorities and the infection prevention/healthcare-epidemiology team at affiliated hospitals. | The local health department and the infection preventionist at the affiliated children’s hospital are notified. You learn that two cases of norovirus have been identified in children who are hospitalized. The parents of those children disclose that they are staying at RMHC® facility. |
| 4 | Investigate | Begin contact tracing to document interactions between ill and asymptomatic patients. [Contact tracing](https://www.cdc.gov/museum/pdf/cdcm-pha-stem-lesson-contact-tracing-lesson.pdf) is the process of identifying people who have recently been in contact with someone diagnosed with an infectious disease.^10^ This will assist with quick identification of new cases. This may also be helpful if preventative treatment is recommended by medical experts. | The local health department suspects that the holiday party may be the source of the norovirus outbreak. RMHC® members create a list that includes everyone who attended or prepared food for the party. Each person who attended the party is asked about symptoms of vomiting and diarrhea. |
| 5 | Communicate | Communicate to staff and guests any recommendations of the public health experts. Tips used by public health experts about [communication during an outbreak](https://www.cdc.gov/eis/field-epi-manual/chapters/Communicating-Investigation.html) may be helpful.^11^ Report all infectious disease outbreaks to RMHC® Global. | The healthcare epidemiologist at the affiliated children’s hospital works with RMHC® team to develop messaging for guests and staff that describe an increased number of cases of GI illness, information about norovirus specific instruction for people with symptoms, and steps healthy people can take to prevent illness. |
| 6 | Liaise | Prepare for potential media coverage and community awareness. Work with RMHC® Global Media Relations on external communications plan. | The facility director works with the communications team at the local health department to respond to an inquiry from a local television station. |
| 7 | Implement | Control measures may include limiting visitation and restricting access to communal areas in the facility. Appendix C provides guidance about when symptomatic guests must leave the facility. Control measures may be different for each kind of infection. | Some states have created toolkits for responding to norovirus outbreaks in [long-term care facilities](https://dphhs.mt.gov/assets/publichealth/CDEpi/CDCPBResources/NorovirusToolkit2019ADA.pdf) or [childcare centers and schools](https://www.cdph.ca.gov/Programs/CID/DCDC/CDPH%20Document%20Library/Norovirus-School-Toolkit.pdf).^12,13^ Control measures for norovirus include restricting sick guests and staff members from the facility until 48 hours after the illness resolves, frequent handwashing with soap and water, and environmental cleaning with a bleach product. |
| 8 | Educate | Create a clear and digestible message to guests about the situation and what you are doing to ensure their safety. Disease-specific information can be found on the Centers for Disease Control and Prevention website (cdc.gov) or from the American Academy of Pediatrics (healthychildren.org). Local and state health departments may be able to assist with messaging. | The communications team, with the assistance of the local health department, creates a print/digital handout about norovirus and prepares a verbal response regarding the infection prevention practices initiated. |
| 9 | Update | Be sure to update local public health authorities with any changes and with outbreak response progress. | Over the next two days, two more guests and one additional staff member develop vomiting and diarrhea. RMHC® staff add their names to the line list and notify the health department. |
| 10 | Decide | Working with local public health authorities, decide when the outbreak has ended and relay this to guests and staff. | In general, an outbreak is over when no new cases have been identified after two incubation periods of the illness. For norovirus, the incubation period is two days. The outbreak might be considered over when no new cases have been identified by at least four days. Ultimately, public health authorities will determine when an outbreak has ended. |

## Table A3. Examples of Significant Varicella Exposures in a Family-Centered Residential Setting

### Chickenpox

- Household: residing in the same household during the contagious period
- Playmate: face-to-face indoor play with an infectious person*
- Multi-family facility: in the same room, family lounge, or common area with an infectious person*

### Zoster (shingles)

- Intimate contact (e.g. touching or hugging) with a person having skin lesions that are deemed contagious.
- Follow the same guidance for chickenpox if the person has disseminated zoster, (e.g., skin lesions in more than one area of the body, usually in patients with weakened immune systems).

* Minimum duration of exposure needed for transmission of the varicella virus is not clearly defined, but may be as short as 5 minutes.

## Table A4. Criteria for Individual to be Considered “Non-Contagious” Despite Exposure to Varicella

Individual must meet at least one of the following criteria (signed medical clearance required):

- History of having chickenpox or shingles (diagnosed by a licensed clinician)
- Documentation of 2 doses of varicella-containing vaccine, separated by at least 3 months
- Documented varicella antibodies (also known as a positive varicella IgG blood test)
- It has been at least 21 days since the person’s last significant exposure to the person with chickenpox or shingles (if the exposed person received post-exposure prophylaxis [intravenous immune globulin (IVIG) or varicella zoster immune globulin (VARIZIG)], it must be at least 28 days since last exposure).

# Appendix B

## Algorithm B1. Symptoms Questionnaire Algorithm (see PDF)

## Algorithm B2. Disease Exposure Questionnaire Algorithm (see PDF)

# Appendix C

## Table C1. Type and Duration of Precautions

### Components of Standard Precautions

| ***Hand hygiene*** | Perform hand hygiene ***before*** preparing food or eating, ***before*** administering medication to a patient and ***after*** changing diapers, toileting or assisting others in toileting, or when hands are visibly dirty. |
| --- | --- |
| ***Glove use*** | Use gloves before contact with blood or body fluids; always perform hand hygiene after glove removal, and never wash and/or reuse the same gloves. |
| ***Respiratory hygiene/cough etiquette*** | Cover coughs and sneezes with a tissue, promptly throw away used tissues, and perform hand hygiene afterwards. Maintain three to six feet (one to two meters) from anyone coughing or sneezing. |
| ***Blood and body fluid precautions*** | Assume that all blood and body fluids may contain contagious germs (e.g., hepatitis viruses, human immunodeficiency virus). |
| ***Safe injection practices*** | Prevent accidental needlesticks and exposure to blood by handling all needles used for injection of medications carefully, not recapping used needles, using needles only once, and disposing used needles separately from all other trash into a rigid, puncture resistant “sharps” container. |
| ***Safe laundry management*** | Do not shake linens and clothing soiled with blood, vomitus, stool, etc. Separate these contaminated items in a plastic bag and wash separately in soap and hot water to prevent transmission of infectious agents. |
| ***Cleaning and disinfection of the environment*** | Create and follow routine housekeeping procedures in each residential facility. All items that require disinfection must first be cleaned with a detergent before using a disinfectant. Toileting and diapering areas must always be separate from food preparation areas. Surfaces and items in common areas and playrooms should be cleaned and disinfected frequently. Toys that are smooth and scrubbable are preferred, because they are easier to wipe down for cleaning and disinfection. |

### Definitions

| ^1^**Exposed, healthy guest** | The “exposed, healthy guest” refers to a non-ill family member of the pediatric patient. The child under medical care is currently diagnosed with an infectious organism/disease. The family member has been “exposed” to the child but is currently healthy. |
| --- | --- |
| **^2^Person with condition** | The “person with condition” can be a family member or a pediatric patient. The person requesting RMH or RMFR programs’ services has been diagnosed with an infectious organism/disease or has developed the specific condition while staying at RMHC® program. |

### Abbreviations

| WMC | Written Medical Clearance Required |
| --- | --- |
| HH | Hand Hygiene Education |
| BBF | Blood & Body Fluid Education |
| RE | Respiratory Etiquette Education |

### Type and Duration of Precautions Recommended for Selected Infections and Conditions

| **Condition** | **Is an exposed, healthy guest^1^ allowed entry?** | **Is a person with condition^2^ allowed entry?** | **Special restrictions within facility** | **Special education handout to family members** | **Comments** |
| --- | --- | --- | --- | --- | --- |
| **Abscess** |  |  |  |  | See “Skin and Soft Tissue Infections” |
| **Acquired Immunodeficiency Syndrome (AIDS)** | Yes | Yes |  | BBF |  |
| **Adenovirus infection** |  |  |  |  | See specific illness for guidance (e.g., conjunctivitis, diarrhea, pneumonia) |
| **Bronchiolitis** |  |  |  |  | See “Respiratory Infection” |
| **Coronavirus, including COVID-19** |  |  |  |  | See “Respiratory Infection” |
| **Campylobacter infection** |  |  |  |  | See “Diarrhea” |
| **Cellulitis** |  |  |  |  | See “Skin and Soft Tissue Infection” |
| ***Chlamydia pneumonia*** |  |  |  |  | See “Respiratory Infection” |
| ***Clostridioides difficile* (“C. diff” diarrhea)** |  |  |  |  | See “Diarrhea” |
| **Conjunctivitis (pink eye)** | | | | | |
| - With active eye drainage or fever | Yes | No |  | HH | See text for details |
| - With no eye drainage | Yes | Yes | Yes, all common areas | HH |  |
| **Cystic Fibrosis (CF)** | Yes | Yes | See comments | RE | CF patients should not socialize. See “Special Populations” for details |
| **Cytomegalovirus (CMV)** | Yes | Yes |  | HH |  |
| **Diarrhea, cause known** | | | | | |
| - Adenovirus | Yes | No, see comments | Yes - Restrict those with diarrhea within the previous 2 weeks from food prep/handling in common kitchen | HH | May enter once fever-free and diarrhea-free for >24 hours |
| - *Campylobacter* | Yes | No, see comments | Yes - Restrict those with diarrhea within the previous 2 weeks from food prep/handling in common kitchen | HH | May enter once fever-free and diarrhea-free for >24 hours |
| - *C difficile* | Yes | No, see comments | Yes - Restrict those with proven *C. difficile* diarrhea within the previous 1 month from food prep/handling in common kitchen | HH | May enter once fever-free and diarrhea-free for >24 hours |
| - *E coli* O157:H7 | Yes | No – WMC |  | HH | See text for details |
| - *E coli* – other types | Yes | No, see comments | Yes - Restrict those with diarrhea within the previous 2 weeks from food prep/handling in common kitchen | HH | May enter once fever-free and diarrhea-free for >24 hours |
| - Enterovirus | Yes | No, see comments | Yes - Restrict those with diarrhea within the previous 2 weeks from food prep/handling in common kitchen | HH | May enter once fever-free and diarrhea-free for >24 hours |
| - Norovirus | Yes | No | Yes - Restrict those with diarrhea within the previous 2 weeks from food prep/handling in common kitchen |  | Highly contagious |
| - Rotavirus | Yes | No | Yes - Restrict those with diarrhea within the previous 2 weeks from food prep/handling in common kitchen |  | Spread within families is common |
| - *Salmonella*, non-typhoid | Yes | No, see comments | Yes - Restrict those with diarrhea within the previous 2 weeks from food prep/handling in common kitchen | HH | May enter once fever-free and diarrhea-free for >24 hours |
| - *Salmonella*, typhoid | Yes | No –WMC |  | HH | See text for details |
| - *Shigella* | Yes | No – WMC |  | HH | See text for details |
| - Viral (not covered elsewhere) | Yes | No, see comments | Yes - Restrict those with diarrhea within the previous 2 weeks from food prep/handling in common kitchen | HH | May enter once fever-free and diarrhea-free for >24 hours |
| - Diarrhea, cause unknown | Yes | No, see comments | Yes - Restrict those with diarrhea within the previous 2 weeks from food prep/handling in common kitchen | HH | May enter once fever-free and diarrhea-free for >24 hours |
| **Diphtheria** | No-WMC | No-WMC |  |  | See text for details |
| **Enterovirus** |  |  |  |  | See Specific Illness (Diarrhea, Hand-Foot-Mouth, Meningitis) |
| **Epstein Barr Virus (EBV)** | Yes | No, see comments |  |  | May enter once fever-free for >24 hours |
| **Hand, foot, mouth disease** | Yes | No, see comments |  |  | May enter once fever-free for >24 hours |
| ***Haemophilus influenzae,* type b (Hib) disease** | Yes | No, see comments |  |  | May enter after 24 hours antibiotics *and* well enough for hospital discharge Family members should be evaluated for post-exposure antibiotics. See text for details. |
| **Hepatitis A** | Yes, only if vaccinated | No –WMC |  | HH | See text for details |
| **Hepatitis B** | Yes | Yes |  | BBF |  |
| **Hepatitis C** | Yes | Yes |  | BBF |  |
| **Herpes simplex virus (HSV)** | | | | | |
| - HSV cold sore (lip) only | Yes | Yes, if certain criteria met. See text for details | Yes, all common areas until cold sore is completely crusted and dry | HH | See text for details |
| - HSV encephalitis | Yes | Yes |  |  |  |
| - HSV rash (skin, eye) | Yes | No - WMC | Yes, all common areas until rash is completely crusted and dry | HH | May enter if certain criteria are met. See text for details |
| **Human immunodeficiency virus (HIV)** | Yes | Yes |  | BBF |  |
| **Human metapneumovirus** |  |  |  |  | See “Respiratory Infection” |
| **Impetigo** |  |  |  |  | See “Skin and Soft Tissue Infections” |
| **Influenza virus** |  |  |  |  | See “Respiratory Infection” |
| **Kawasaki disease** | Yes | Yes |  |  |  |
| **Lice** | Yes | No |  |  | Family members should have scalp evaluation for lice infestation |
| **Listeriosis *(Listeria monocytogenes)*** | Yes | Yes |  |  |  |
| **Lyme disease** | Yes | Yes |  |  |  |
| **Mpox (previously *monkeypox*)** | Yes, see comments | No-WMC |  |  | Collaboration with local public health partners is recommendation. See text for details. |
| **Malaria** | Yes | Yes |  |  |  |
| **Measles** | No, WMC | No, WMC |  |  | See text for details |
| **Meningitis, bacterial** | Yes | No, see comments |  |  | May enter after 24 hours antibiotics *and* well enough for hospital discharge. Family members should be evaluated for post-exposure antibiotics. See text for details. |
| **Meningitis, viral** | Yes | No, see comments |  |  | May enter once fever-free *and* diarrhea-free for >24 hours |
| **Meningococcal disease *(Neisseria meningitidis)*** | Yes | No, see comments |  |  | May enter after 24 hours antibiotics *and* well enough for hospital discharge. Family members should be evaluated for post-exposure antibiotics. See text for details. |
| **Methicillin Resistant *Staphylococcus aureus* (MRSA) – active infection** |  |  |  |  | See specific illness for guidance (e.g., skin infection, pneumonia) |
| **MRSA colonization (person has a history of MRSA infection, but is currently healthy)** | Yes | Yes |  | HH | See “Multi-Drug Resistant Bacteria (MDRO) text for details. |
| **Molluscum contagiosum** | Yes | Yes |  | HH |  |
| **Mononucleosis (“mono”)** |  |  |  |  | Refer to “Epstein Barr Virus” |
| **Multi-Drug Resistant Organism (MDRO) – active infection** |  |  |  |  | See specific illness for guidance (e.g., skin infection, pneumonia) |
| **MDRO colonization (person has a history of MDRO infection, but is currently healthy)** | Yes | Yes |  | HH | See “Multi-Drug Resistant Bacteria (MDRO) text for details. |
| **Mumps** | No, WMC | No, WMC |  |  | See text for details |
| ***Mycoplasma* pneumonia** |  |  |  |  | See “Respiratory Diseases” |
| **Necrotizing enterocolitis (NEC)** | Yes | Yes, see comments |  |  | May enter once well enough for hospital discharge |
| **Norovirus** |  |  |  |  | See “Diarrhea” |
| **Parainfluenza Virus** |  |  |  |  | See “Respiratory Diseases” |
| **Parvovirus B19** | Yes | No, see comments |  |  | May enter once fever-free for >24 hours |
| **Pertussis** | Yes, see comments | No-WMC |  |  | Exposed person should be evaluated for exposure antibiotics. See text for details. |
| **Pinworm** | Yes | Yes |  | HH |  |
| **Pneumonia** |  |  |  |  | See “Respiratory Infection” |
| **Respiratory syncytial virus (RSV)** |  |  |  |  | See “Respiratory Infection” |
| **Respiratory infection** | | | | | |
| - Adenovirus | Yes | No, see comments | Yes, all common areas while symptomatic. See text for details | HH, RE | May enter once fever-free for >24 hours |
| - Bacterial Pneumonia | Yes | No, see comments |  | HH, RE | May enter after 24 hours of antibiotics *and* fever-free for >24 hours |
| - COVID-19 | Yes | No, see comments | Yes, all common areas while symptomatic. See text for details | HH, RE | May enter once fever-free for >24 hours |
| - *Chlamydia* | Yes | No, see comments | Yes, all common areas while symptomatic. See text for details | HH, RE | May enter after 24 hours of antibiotics *and* fever-free for >24 hours |
| - Enterovirus | Yes | Yes | Yes, all common areas while symptomatic. See text for details | HH, RE | May enter once fever-free for >24 hours |
| - Fungal | Yes | Yes |  | RE | Not spread from person-to-person |
| - Influenza virus | Yes | No, see comments | Yes, all common areas while symptomatic. See text for details | HH, RE | May enter once fever-free for >24 hours |
| - *Legionella* | Yes | Yes |  | RE | Not spread from person-to-person |
| - *Mycoplasma* | Yes | No, see comments | Yes, all common areas while symptomatic. See text for details | HH, RE | May enter after 24 hours of antibiotics *and* fever-free for >24 hours |
| - Parainfluenza virus | Yes | No, see comments | Yes, all common areas while symptomatic. See text for details | HH, RE | May enter once fever-free for >24 hours |
| - RSV | Yes | No, see comments | Yes, all common areas while symptomatic. See text for details | HH, RE | May enter once fever-free for >24 hours |
| - Rhinovirus | Yes | No, see comments | Yes, all common areas while symptomatic. See text for details | HH, RE | May enter once fever-free for >24 hours |
| - Viral, not covered elsewhere | Yes | No, see comments | Yes, all common areas while symptomatic. See text for details | HH, RE | May enter once fever-free for >24 hours |
| **Rheumatic fever** | Yes | Yes |  |  |  |
| **Ringworm** | Yes | Yes |  |  |  |
| **Rotavirus infection** |  |  |  |  | See “Diarrhea” |
| ***Salmonella* infection** |  |  |  |  | See “Diarrhea” |
| **Scabies** | Yes | No |  |  | See text for details |
| ***Shigella* infection** |  |  |  |  | See “Diarrhea” |
| **Skin, soft tissue infections** | | | | | |
| - Actively draining abscess | Yes | No |  | HH |  |
| - Abscess with no drainage | Yes | Yes |  | HH | Abscess must be completely covered and contained with a dry dressing |
| - Cellulitis or Impetigo | Yes | Yes |  | HH |  |
| - Mastitis | Yes | Yes |  | HH |  |
| ***Staph aureus* infection** |  |  |  |  | See specific illness |
| **Strep infection** | | | | | |
| - Group A strep (e.g., Strep throat) | Yes | No, see comments |  |  | May enter after 24 hours of antibiotics *and* fever-free for >24 hours |
| - Group B strep | Yes | Yes |  |  |  |
| - Not group A or B | Yes | Yes |  |  |  |
| **Toxic Shock Syndrome** | Yes | Yes, see comments |  |  | May enter after 24 hours antibiotics *and* well enough for hospital discharge |
| **Tuberculosis** | No, WMC | No, WMC |  |  | See text for details |
| **Urinary tract infection (UTI)** | Yes | Yes |  |  |  |
| **Varicella Zoster** | | | | | |
| - Chickenpox | No, WMC | No, WMC |  |  | May enter if certain criteria met. See text for details |
| - Shingles (Zoster) | No, WMC | No, WMC | Yes, all common areas until rash is completely crusted and dry | HH | May enter if certain criteria met. See text for details |
| **Viral respiratory infection** |  |  |  |  | See ”Respiratory Infection” |
| **Whooping cough** |  |  |  |  | See “Pertussis” |
| **Wound infection** |  |  |  |  | See “Skin and Soft Tissue Infection” |
| **Zoster** |  |  |  |  | See “Varicella Zoster” |

## Table C2. Frequently encountered special populations in family-centered residential facilities

| **Newborn infants**, especially infants born prematurely | All infants are born with an immature immune system:   - An infants’ immune system develops during the first months of life - The more premature an infant is, the weaker the immune system is at birth - Prematurely born infants with chronic lung disease or certain genetic conditions may be at higher risk of acquiring infections and developing serious complications |
| --- | --- |
| **Individuals living with immune deficiencies** (weakened immune systems), e.g.:   - Congenital (born with a weakened immune system) - Acquired (e.g., HIV) | Some children are born with immune deficiencies or complete absence of various components of their immune systems, called congenital immunodeficiencies:   - Congenital immunodeficiencies can be so severe that a child may eventually require a bone marrow or stem cell transplant - Most congenital immune deficiencies result from genetic abnormalities and can affect different components of the immune system   After birth, children may acquire immunodeficiency for many reasons, including HIV infection or loss of spleen function due to disease (e.g., sickle cell disease) or surgical spleen removal |
| **Individuals undergoing immune-suppressive therapy**, e.g.:   - Cancer chemotherapy - Hematopoietic stem cell transplant (HSCT, also referred to as bone marrow transplant, or BMT) - Solid organ transplant (e.g., lung, liver, kidney, heart transplant) - Rheumatologic diseases (e.g., systemic lupus erythematosus (SLE), juvenile idiopathic arthritis (JIA) - Inflammatory bowel disease (e.g., Crohn’s disease, ulcerative colitis) | - Potent chemical and biological agents or radiation that suppress the body’s immune system are treatments for a variety of conditions (e.g., cancer, systemic lupus erythematosus (SLE), inflammatory bowel disease, etc.). - Medical professionals can identify the degree of immune suppression for the stage of a patient’s treatment. Stem cell transplant patients are the most vulnerable, usually within the first 100 days after transplant or during all periods when they experience rejection of the transplanted cells (graft vs. host disease). These patients are especially at risk of infections caused by mold normally found in the air, particularly near construction sites or water leaks. The healthcare team can provide specific safety recommendations about immune compromised House guests, to include sometimes separating these patients from others until their immunity improves. |
| **Individuals living with cystic fibrosis** | - Patients living with CF are at high risk for serious lung infections - These individuals will develop worsening lung function after infections with common cold viruses. - Patients with CF require additional protection compared to other patients in order to prevent transmission from contaminated respiratory therapy equipment and from respiratory secretions of other patients with CF. - In residential houses, siblings who live together in the same home may reside and socialize together. However, non-siblings with CF should not socialize, have meals, or room together.^14^ An updated *Guideline for Infection Prevention and Control in People with Cystic Fibrosis* was released in 2013.^14^ - Although patients with CF have traditionally found support by socializing with other patients with CF, the threat of acquiring dangerous bacteria from one another has led to recommendations that patients with CF *should not* socialize with each other. - Bacteria such as *Burkholderia cepacia* complex (BCC) and *Pseudomonas aeruginosa* can be especially dangerous for individuals with CF.^14^ Person-to-person transmission of BCC has been demonstrated among children and adults with CF in healthcare settings, during social contacts, and among siblings with CF.^14^ |
| **Persons who are pregnant** | - Pregnancy is not associated with a true immune deficiency, but certain infections can affect the fetus adversely. - Pregnant persons should:   - Have consistent hand hygiene   - Avoid sick contacts, cat litter, and undercooked meat   - Prior to arrival at a residential facility, be counseled by their obstetricians about precautions when staying in the residential facility |

# References

1. Guzman-Cottrill JA, Bryant KA, Zerr DM, et al. Infection prevention and control guidance for Ronald McDonald Houses: a needs assessment. *Infect Control Hosp Epidemiol*. Mar 2012;33(3):299-301. doi:10.1086/664054

2. World Health Organization. WHO recommendations for routine immunication - summary tables. <https://www.who.int/teams/immunization-vaccines-and-biologicals/policies/who-recommendations-for-routine-immunization---summary-tables>

3. Centers for Disease Control and Prevention. Immunization schedules. <https://www.cdc.gov/vaccines/schedules/index.html>

4. Centers for Disease Control and Prevention. Food Safety Home Page. Accessed July 1, 2023, <https://www.cdc.gov/foodsafety/index.html>

5. US Food and Drug Administration, Center for Food Safety and Applied Nutrition. Food safety in the kitchen. Accessed July 1, 2023, <https://www.fda.gov/food/buy-store-serve-safe-food/food-safety-your-kitchen>

6. US Department of Health and Human Services, FoodSafety.gov. Cook to a safe minimum internal temperature. Accessed July 1, 2023, <https://www.foodsafety.gov/food-safety-charts/safe-minimum-internal-temperatures>

7. US Department of Health and Human Services, FoodSafety.gov. Cold food storage chart. Accessed July 1, 2023, <https://www.foodsafety.gov/food-safety-charts/cold-food-storage-charts>

8. Centers for Disease Control and Prevention. Norovirus outbreaks. Accessed October 27, 2023, <https://www.cdc.gov/norovirus/outbreaks/index.html>

9. Centers for Disease Control and Prevention. Line list template. Accessed October 27, 2023, <https://www.cdc.gov/urdo/downloads/linelisttemplate.pdf>

10. Centers for Disease Control and Prevention. Contact tracing. <https://www.cdc.gov/museum/pdf/cdcm-pha-stem-lesson-contact-tracing-lesson.pdf>

11. Tumpey AJ, Daigle D, Nowak G. Communicating during an outbreak or public health investigation. CDC Field Epidemiology Manual. Accessed October 27, 2023, <https://www.cdc.gov/eis/field-epi-manual/chapters/Communicating-Investigation.html>

12. Montana Department of Public Health and Human Services. Norovirus Outbreaks in Long Term Care Facilities. Accessed October 27, 2023, <https://dphhs.mt.gov/assets/publichealth/CDEpi/CDCPBResources/NorovirusToolkit2019ADA.pdf>

13. Infectious Diseases Branch, California Department of Public Health. Norovirus Toolkit for School and Childcare Center Outbreaks. Accessed October 27, 2023, <https://www.cdph.ca.gov/Programs/CID/DCDC/CDPH%20Document%20Library/Norovirus-School-Toolkit.pdf>

14. Saiman L, Siegel JD, LiPuma JJ, et al. Infection prevention and control guideline for cystic fibrosis: 2013 update. *Infect Control Hosp Epidemiol*. Aug 2014;35 Suppl 1:S1-S67. doi:10.1086/676882
